# Author Correction: Temporal changes in the gene expression heterogeneity during brain development and aging

**DOI:** 10.1038/s41598-023-37105-0

**Published:** 2023-06-22

**Authors:** Ulaş Işıldak, Mehmet Somel, Janet M. Thornton, Handan Melike Dönertaş

**Affiliations:** 1 Department of Biological Sciences, Middle East Technical University, 06800 Ankara, Turkey; 2 European Molecular Biology Laboratory, European Bioinformatics Institute, Wellcome Trust Genome Campus, Hinxton, Cambridge, CB10 1SD UK

Correction to: *Scientific Reports* 10.1038/s41598-020-60998-0, published online 05 March 2020

The original version of this Article contained errors.

In Figure 2c, the legend of the graph "Change in Heterogeneity" was incorrectly given as "Change in Expression".

The original Figure 2 and accompanying legend appear below.

**Figure 2:**
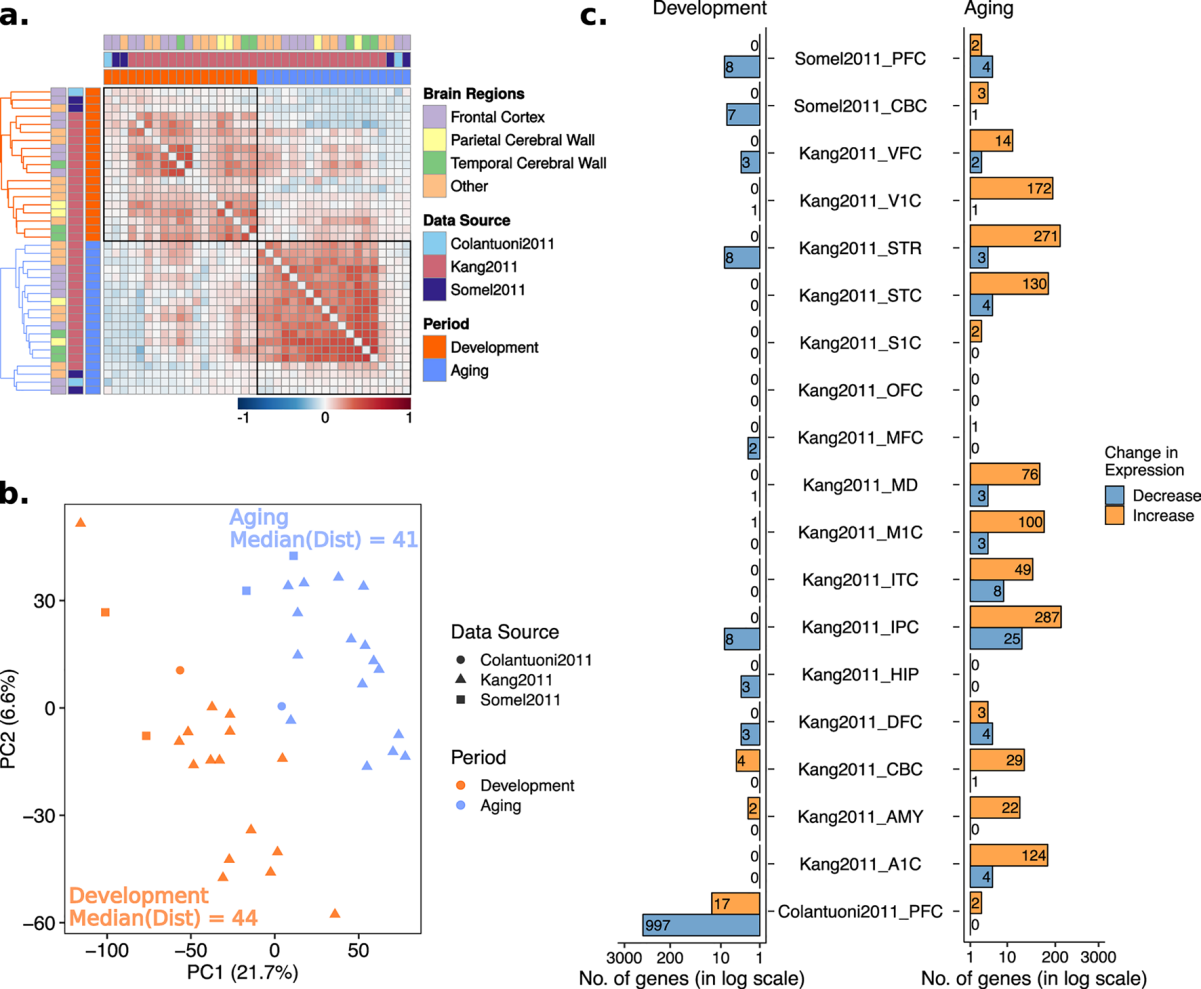
Age-related change in gene expression heterogeneity during development and aging. The procedures are similar to those in Fig. 1, except, age-related heterogeneity changes (ρ values) were used instead of expression changes (β values). (**a**) Spearman correlations among age-related heterogeneity changes (ρ values) across datasets. (**b**) Principal component analysis (PCA) of heterogeneity change with age. (**c**) The number of genes showing significant heterogeneity change in aging and development.

In addition, in the Supplementary Data folder, the list of outliers given in Supplementary Table S1 was omitted.

The original Supplementary Table S1 file is provided below.

The original Article and accompanying Supplementary Table S1, within the Supplementary Data folder, have been corrected.

## Supplementary Information

Supplementary Table S1.

