# Supplementary material for: Temporal changes in the gene expression heterogeneity during brain development and aging
Source: Sci Rep. 2020 Mar 5;10:4080. doi: 10.1038/s41598-020-60998-0 (PMC7058021; doi:10.1038/s41598-020-60998-0)

Temporal changes in the gene expression heterogeneity during brain development and aging

Ulaş Işıldak[1], Mehmet Somel[1], Janet M. Thornton[2], Handan Melike Dönertaş[2*]

[1]Department of Biological Sciences, Middle East Technical University, 06800, Ankara, Turkey.
[2]European Molecular Biology Laboratory, European Bioinformatics Institute, Wellcome Trust Genome Campus, Hinxton, Cambridge, CB10 1SD, UK.
*correspondence: donertas.melike@gmail.com

Supplementary Tables

Table S1: The list of human brain gene expression datasets analyzed in the study.

Table S2: Genes showing consistent heterogeneity increase (n = 147) across all 19 datasets and the trajectory clusters they belong to.

Table S3: KEGG Pathways that are significantly associated (FDR corrected $p \leq 0.1$) with consistent heterogeneity change in aging.

Table S4: Gene Ontology Biological Process Categories that are significantly associated (FDR corrected $p \leq 0.1$) with consistent heterogeneity change in aging.

Table S5: Gene Ontology Molecular Function Categories that are significantly associated (FDR corrected $p \leq 0.1$) with consistent heterogeneity change in aging.

Table S6: Gene Ontology Cellular Component Categories that are significantly associated (FDR corrected $p \leq 0.1$) with consistent heterogeneity change in aging.

Table S7: Reactome Pathways that are significantly associated (FDR corrected $p \leq 0.1$) with consistent heterogeneity change in aging.

Table S8: Transcription factors that are significantly associated (FDR corrected $p \leq 0.1$) with consistent heterogeneity change in aging.

Table S9: miRNAs that are significantly associated (FDR corrected $p \leq 0.1$) with consistent heterogeneity change in aging.

Table S10: miRNAs that are significantly associated (FDR corrected $p \leq 0.1$) with consistent heterogeneity change in development.

Table S11: GSEA results showing the association between the aging-related traits (Johnson et al. 2015) and consistent heterogeneity change in aging.

Supplementary Figures

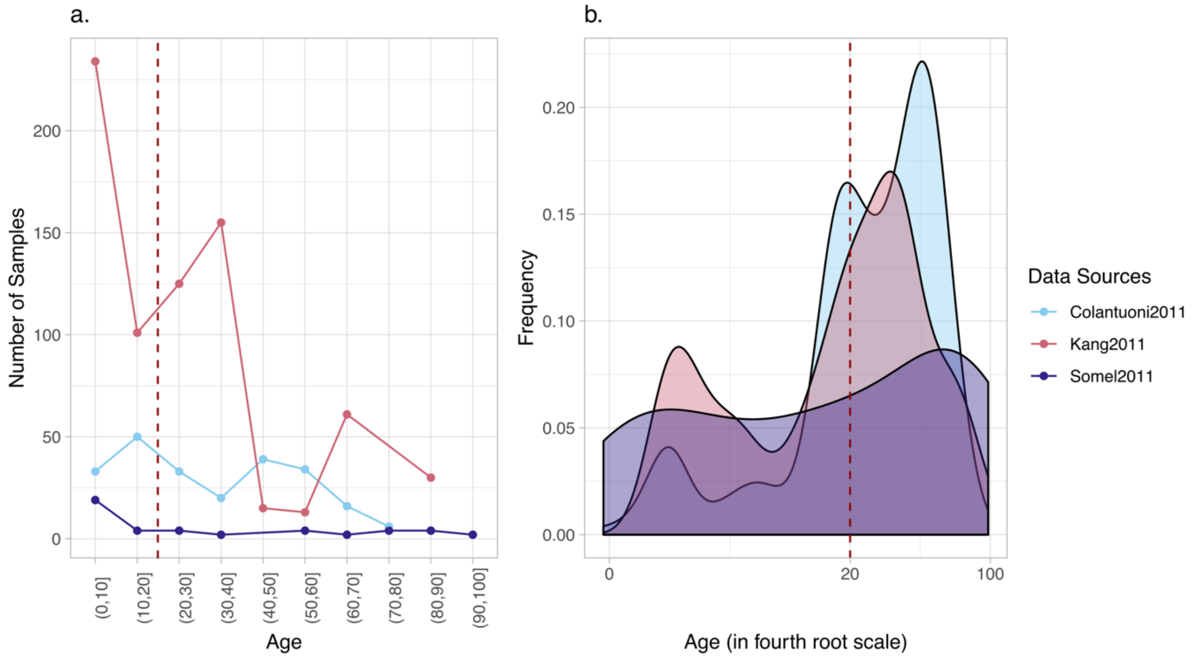

Figure S1: The distribution of ages for the samples used in the analysis. (a) Scatter plot showing number of samples (x-axis) included in age intervals (y-axis). (b) Distribution of samples in fourth root scale of ages. The horizontal dotted lines reflect the separation point of development and aging (age of 20). Data sources are indicated with different colors.

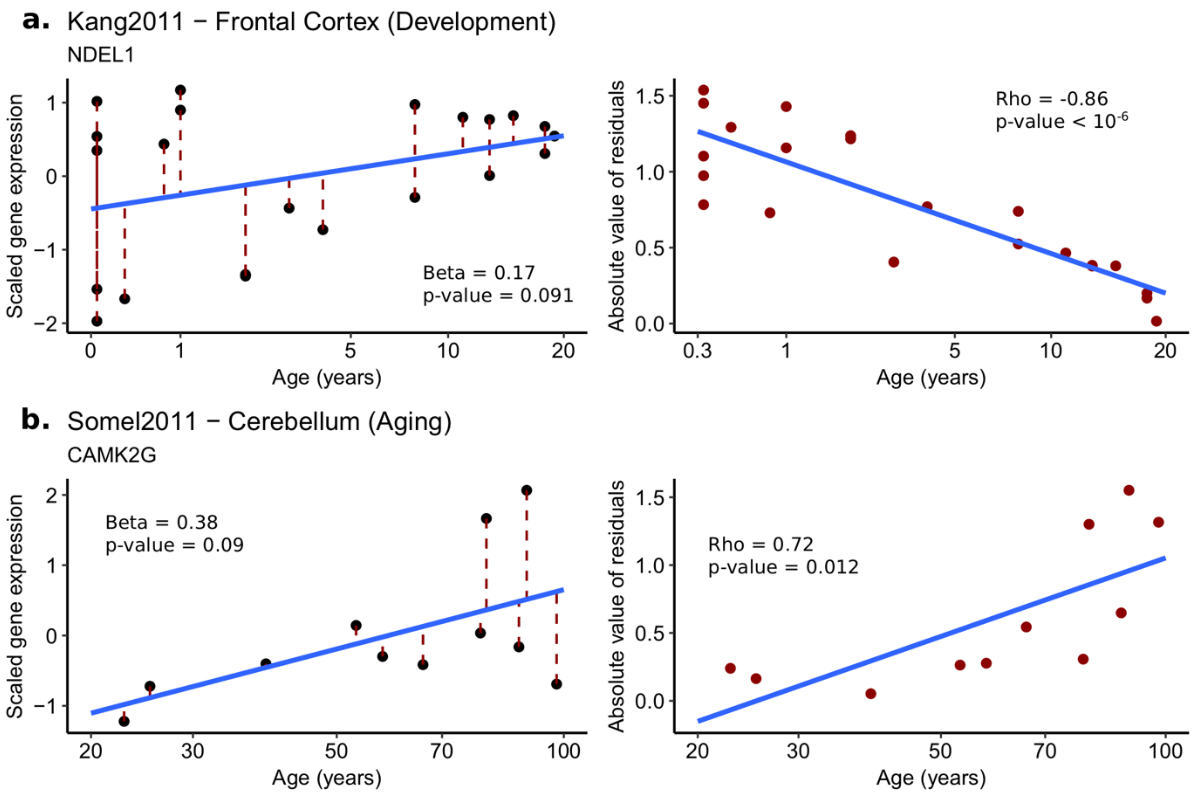

Figure S2: Demonstration of the method used to calculate age-related changes in expression level and heterogeneity of two genes during development (a) and aging (b). *Left panels:* Age-related expression change is quantified by a linear model using scaled gene expression values (y-axis) and the fourth root of ages (x-axis). Beta values and raw *p*-values are indicated on the figure. The blue line is drawn based on the linear model, and the red dotted lines represent residuals. Right panels: corresponding scatterplots for the absolute values of residuals (y-axis) and fourth root of age (x-axis). Rho and raw *p*-values are calculated based on Spearman's correlation test. The blue line is drawn based on a linear model between the absolute value of residuals and the fourth root of age, only for demonstration purposes.

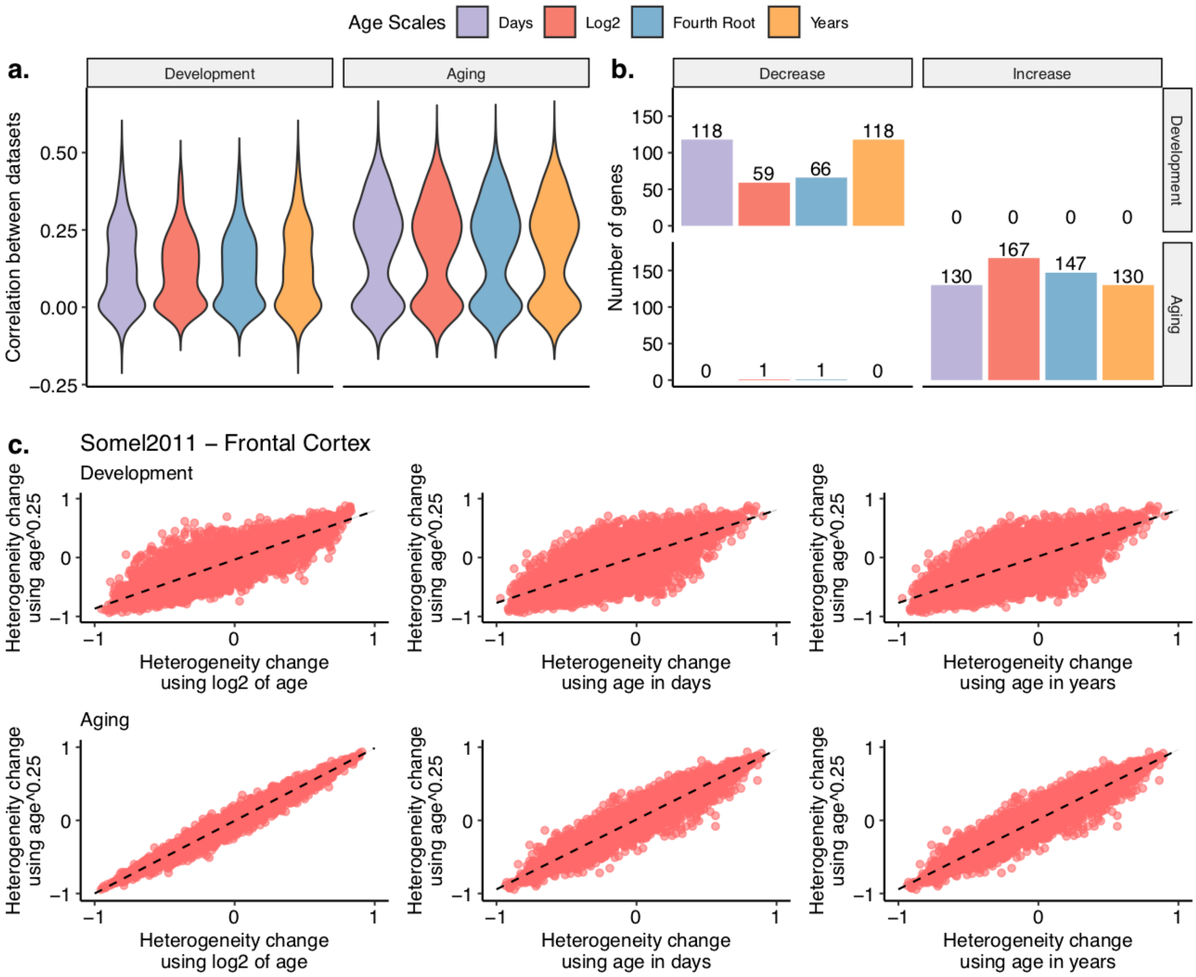

Figure S3: Different age scales yields consistent results. We performed downstream analyses using age in days, age in years, log2 of age in days, and fourth root of age in days. (a) Distribution of correlation coefficients between age-related heterogeneity changes across development and aging datasets (y-axis), using different age scales (x-axis). (b) The number of genes showing consistent heterogeneity change across all 19 datasets (y-axis), using different age scales. (c) Scatterplots of age-related heterogeneity change values of 11,137 genes from one example dataset (Somel2011_PFC) calculated using the fourth root of age in days (y-axis) and different scales including log2 of age in days, age in days and age in years (x-axis) during development (upper panel) and aging (lower panel).

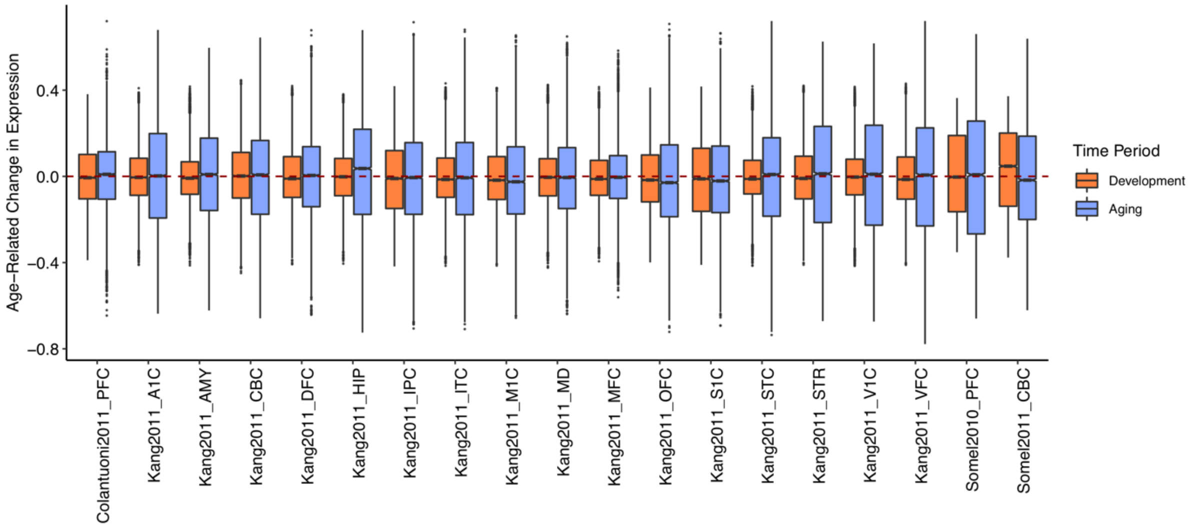

Figure S4: Distribution of age-related change in expression values (Beta values) for each dataset (x-axis) during development and aging. Beta values (y-axis) were computed by linear models using scaled expression values and the fourth root of age for each gene in each dataset during development (orange) and aging (blue) separately (see Figure S2).

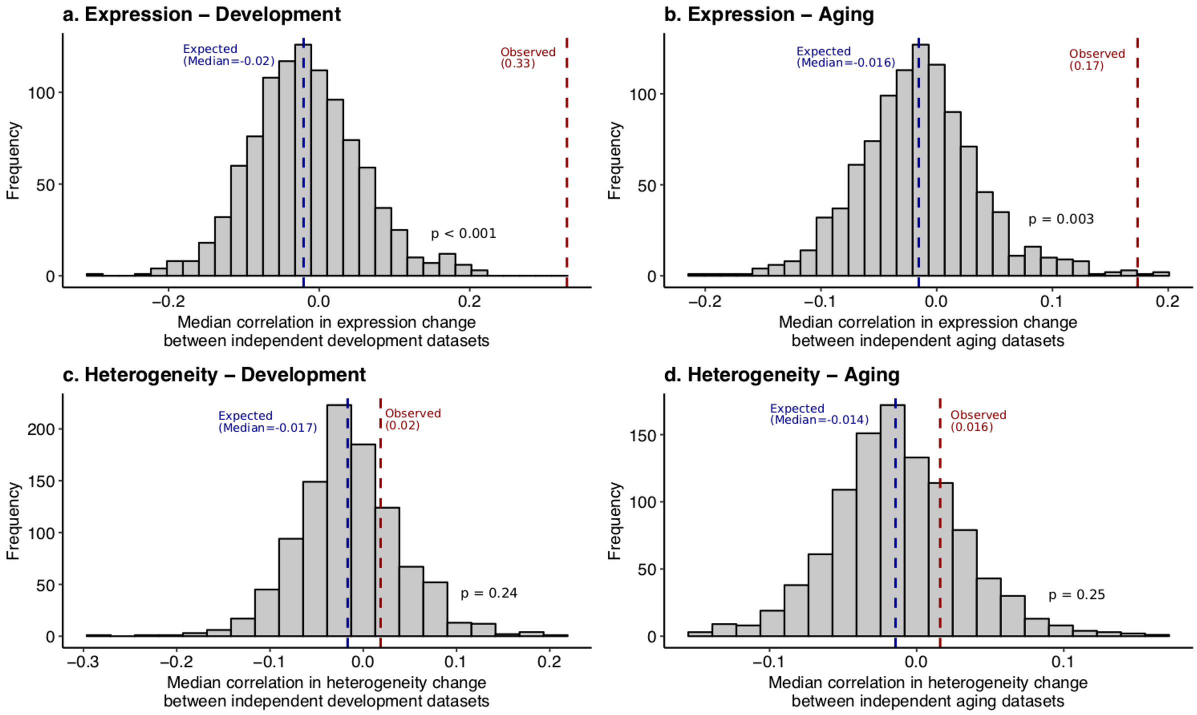

Figure S5: Permutation test results for dataset correlations of expression and heterogeneity change during development and aging. We constructed the distributions by calculating median correlation between all possible three independent datasets from three data sources for all permutations. Similarly, the observed values were calculated as a median value for all three independent dataset combinations from three data sources (i.e. the median value of: Median Correlation(Kang2011_A1C, Somel2011_PFC, Colantuoni2011_PFC), Median Correlation(Kang2011_AMY, Somel2011_CBC, Colantuoni2011_PFC), …). (a, b) Permutation test results for the significance of observed correlation in gene expression change during development (a) and aging (b). (c, d) Permutation test results for the significance of observed correlation in heterogeneity change during development (c) and aging (d).

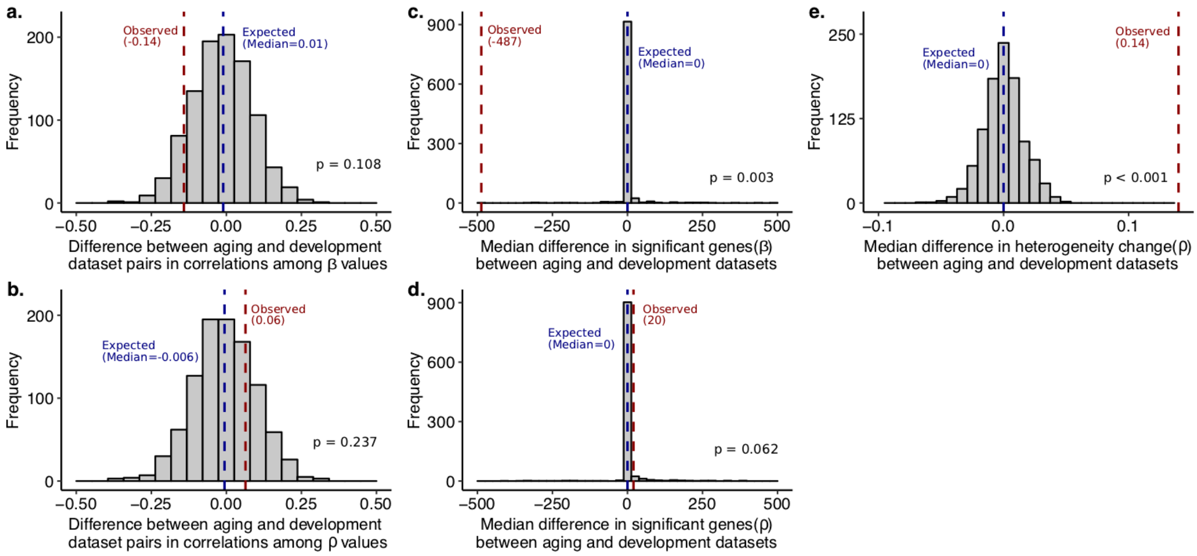

Figure S6: (a, b) Permutation test results for differences between dataset correlations during development and aging. Distributions were constructed using the median differences between correlations among aging datasets and development datasets for 1000 permutations, i.e. negative values imply higher correlation among development datasets compared to aging datasets, while positive values indicate higher correlation during aging. (a) Distribution of median differences between correlations among age-related expression changes (β values) among aging datasets and development datasets. (b) The same distribution for heterogeneity changes (ρ values). (c, d) Permutation test results for the median difference in the number of genes showing significant change between aging and development datasets, in terms of (c) expression change and (d) heterogeneity change. For each permutation, *p*-values were corrected for multiple testing using FDR method. (e) Permutation test result for the median heterogeneity change differences between aging and development datasets. Associated *p*-values are from one-tailed tests.

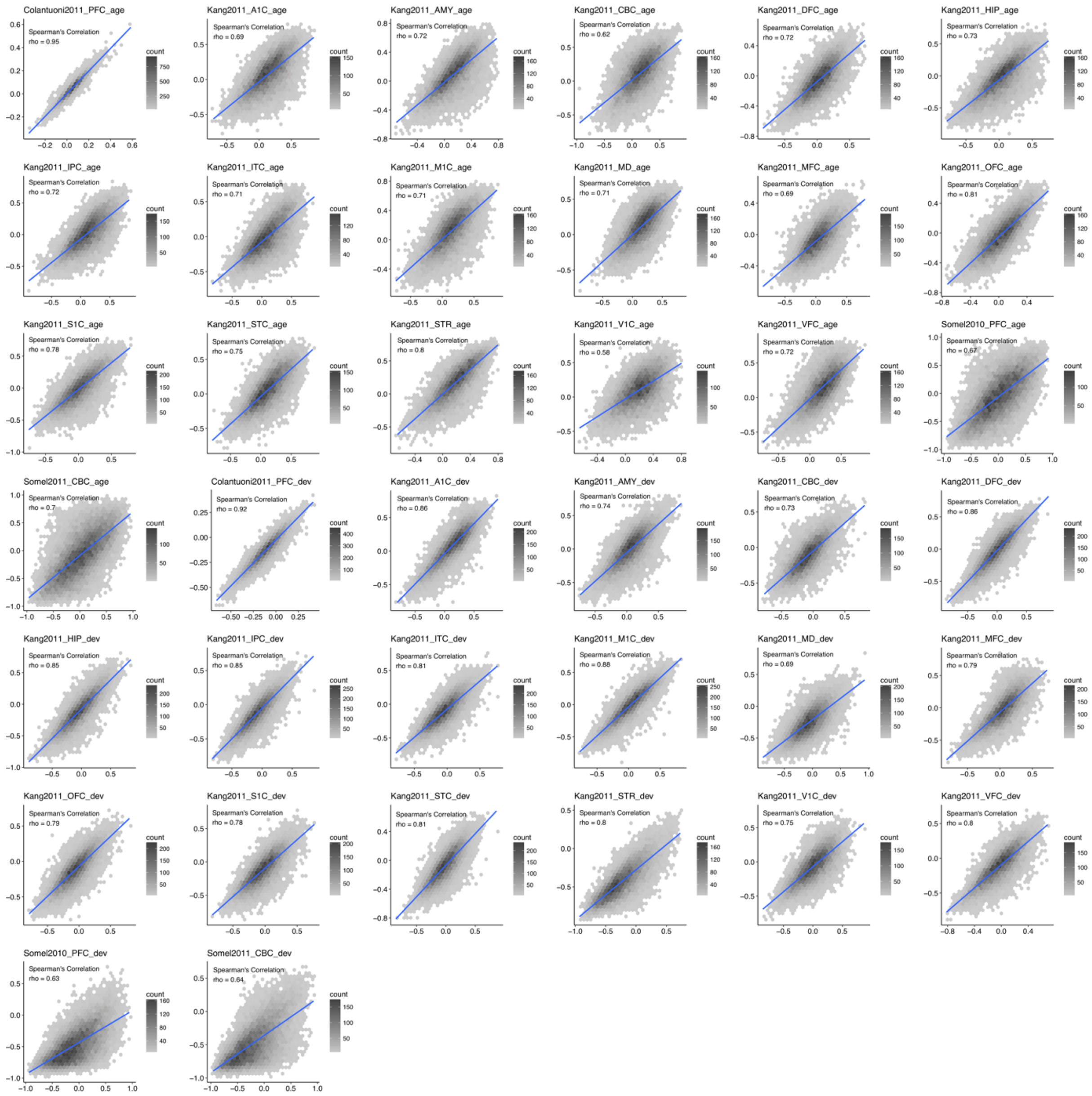

Figure S7: Hexagonal density maps for the changes in heterogeneity based on the residuals from a linear model (x-axis) and loess regressions (y-axis). The color intensity shows the number of genes in that hexagonal bin, where darker color shows more genes. The blue line is drawn using linear regression, for demonstration purposes. The correlations between the values are calculated using Spearman's correlation test.

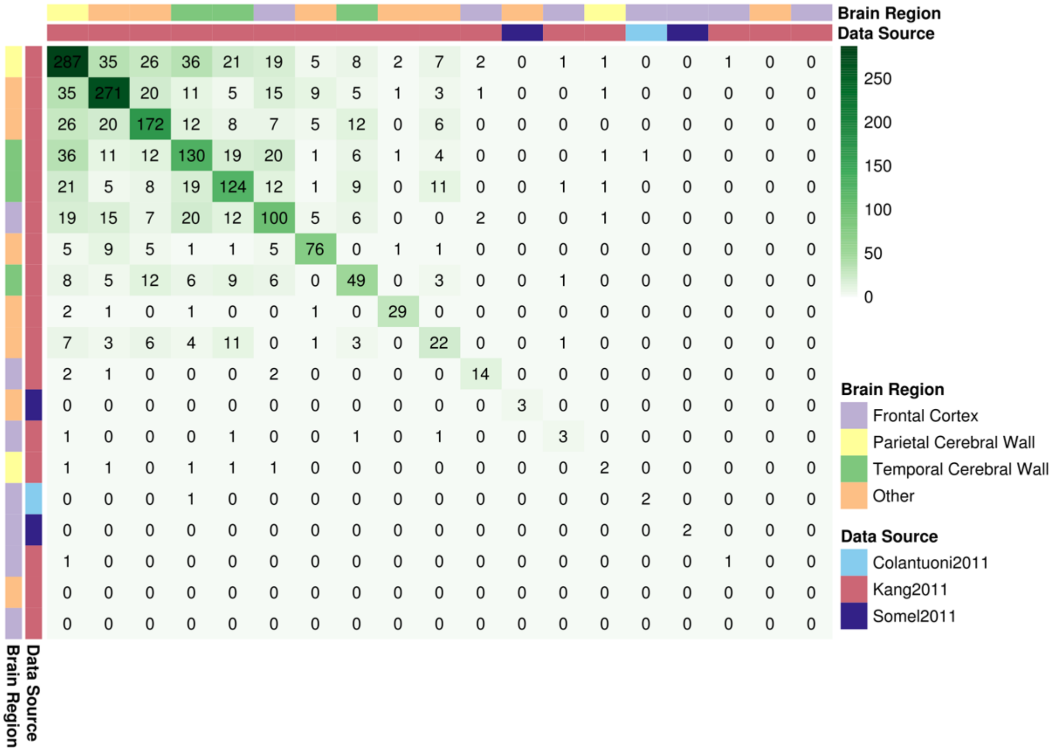

Figure S8: Overlaps between datasets for the genes showing a significant increase in heterogeneity. *p*-values are computed using Spearman correlation test between the absolute value of residuals and fourth root of ages, followed by FDR correction. The color intensity of cells reflects an increased number of overlapping genes among two corresponding datasets, while the numbers show the exact numbers.

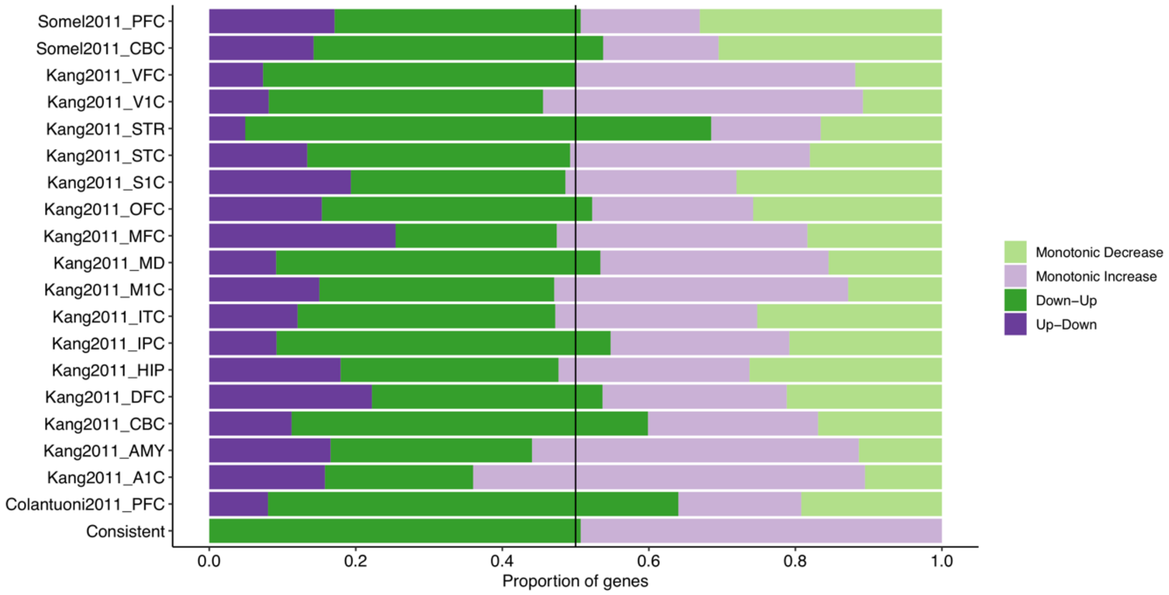

Figure S9: The proportion of different trends in age-related heterogeneity change in each dataset and among the genes showing a consistent increase across aging datasets (n = 147). No effect size or significance cutoff was used. Up-down: increase in development & decrease in aging; down-up: decrease in development & increase in aging; monotonic increase: increase in development and aging; monotonic decrease: decrease in development and aging.

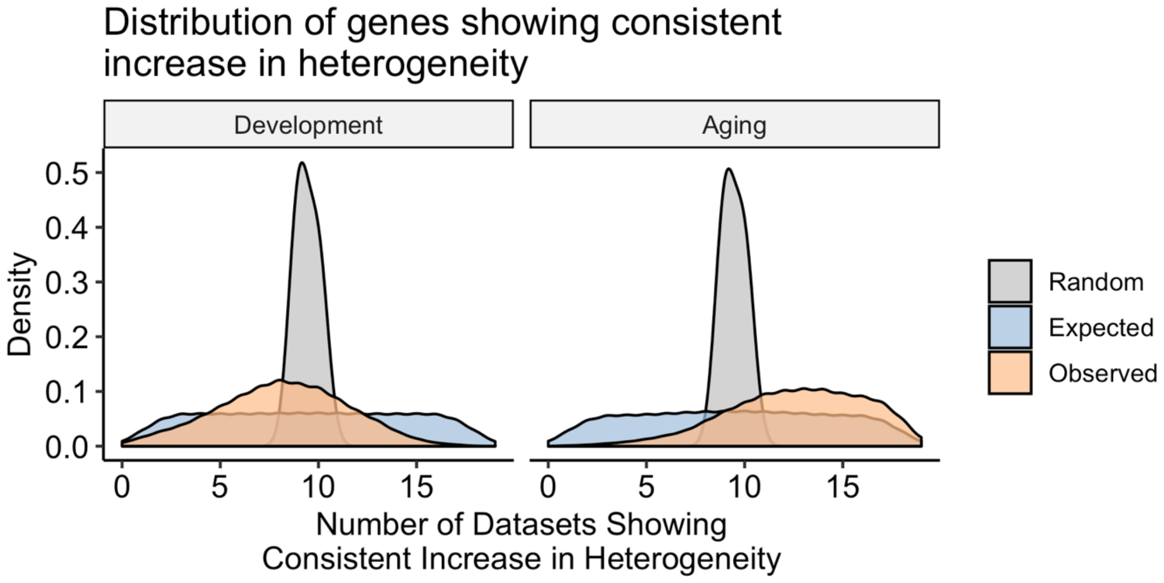

Figure S10: Random (expectation calculated with independent permutations), expected (based on permutations taking dataset dependency into account) and observed consistency in the heterogeneity change across datasets in development and aging. This plot corresponds to Figure 3c, but here we also include the expectation based on independent permutations (‘Random’), and demonstrate that it is less stringent.

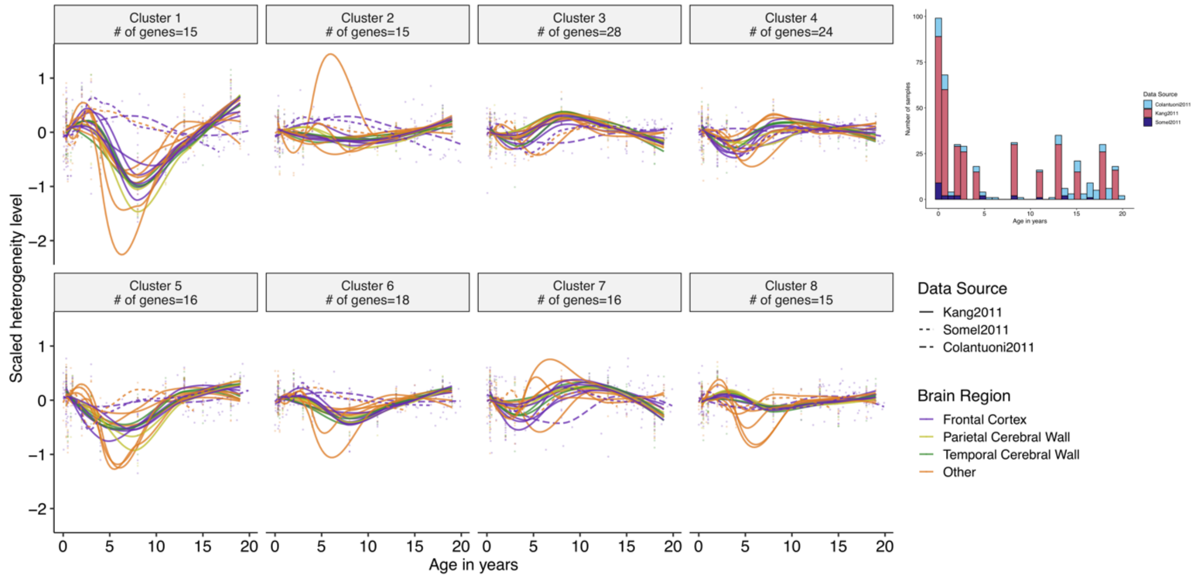

Figure S11: Heterogeneity trajectories in development for the genes that were clustered according to their heterogeneities in aging, using k-means clustering method (in Figure 4). The x-axis shows the age and the y-axis shows scaled heterogeneity level (residuals from linear model). Spline curves represent mean age-related heterogeneity changes of genes in each cluster, from each dataset and brain region. The colors and line-types of curves specify different brain regions and data sources, respectively. Different patterns observed between age of 5 to 8 could be biologically relevant but it is important to note that the number of samples in this age range is low. The age distribution of samples in development datasets is given as a bar plot in upper right corner.

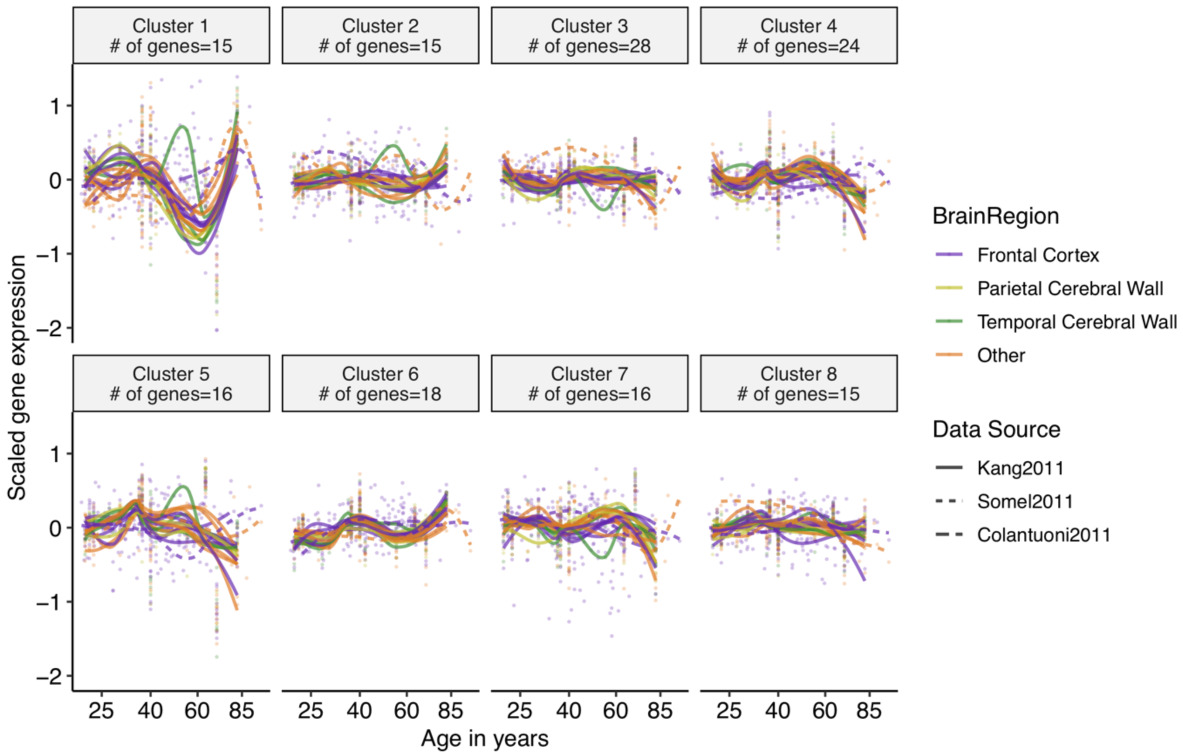

Figure S12: Gene expression trajectories of the genes that were clustered according to their heterogeneities, using k-mean clustering method (in Figure 4). The x-axis shows the age on the fourth root scale, and the y-axis shows scaled gene expression values. Spline curves represent mean age-related expression changes of genes in each cluster, from each dataset and brain region. The colors and line-types of curves specify different brain regions and data sources, respectively.

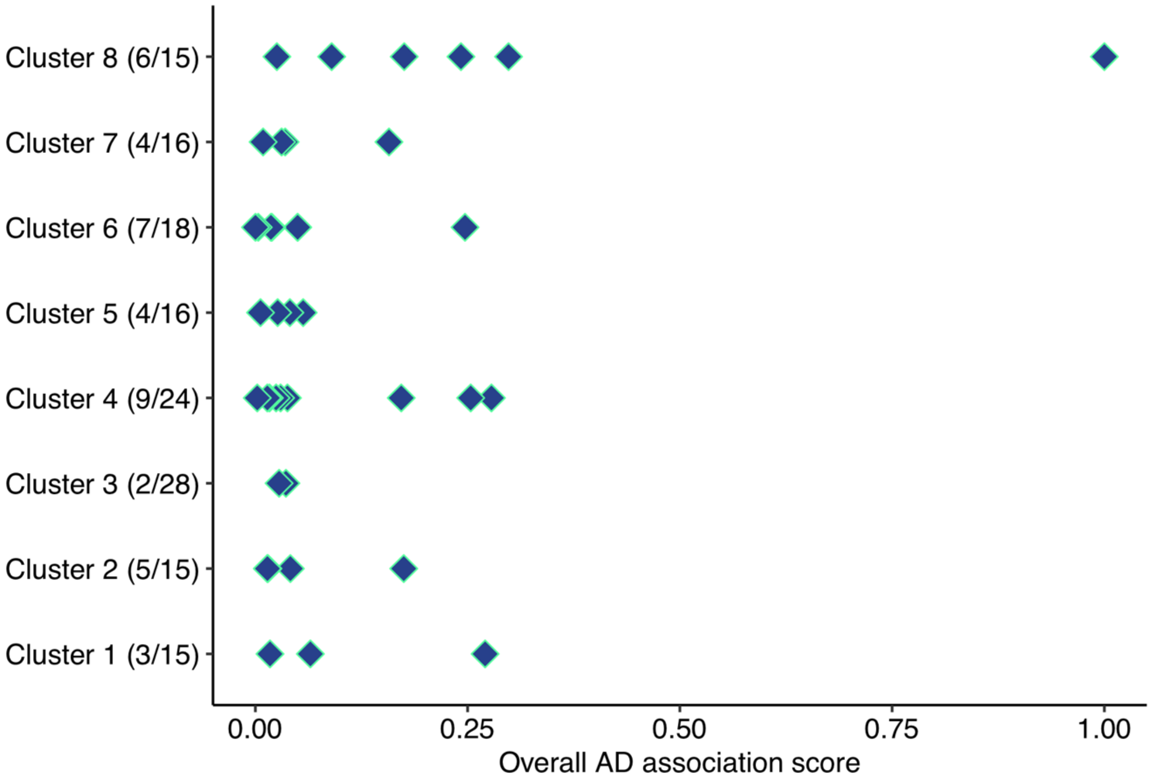

Figure S13: Association between the Alzheimer's-related genes and the genes that consistently become more heterogeneous in aging (*n* = 147) and belong to different heterogeneity trajectories (Figure 4). The x-axis shows overall score for the association with Alzheimer's Disease, while the y-axis shows different clusters in Figure 4. Numbers in the parenthesis on the y-axis reflect the proportion of the genes in clusters found to be in association with AD (40/147, overall).

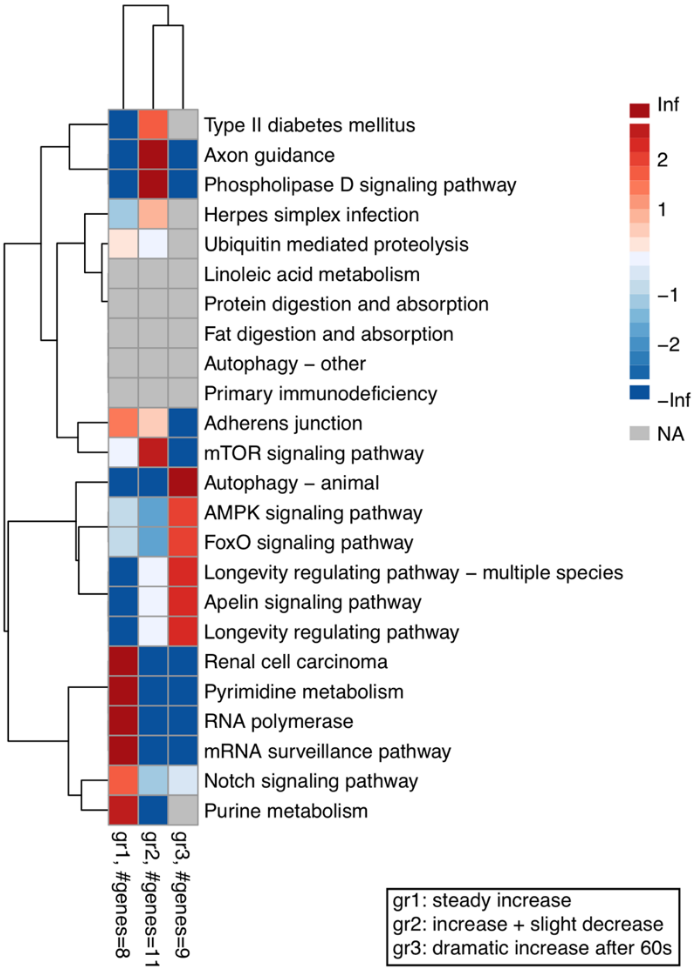

Figure S14: Heatmap showing the association between different heterogeneity trajectories and KEGG pathways that are significantly associated with a consistent change in heterogeneity during aging. Heterogeneity trajectories are based on the definitions in the main text and i) gr1 includes clusters 3 and 7, ii) gr2 includes clusters 4, 5, and 8, and iii) gr3 includes clusters 1, 2, and 6. The colors represent the Odd’s ratio, where red shows enrichment and blue shows depletion.

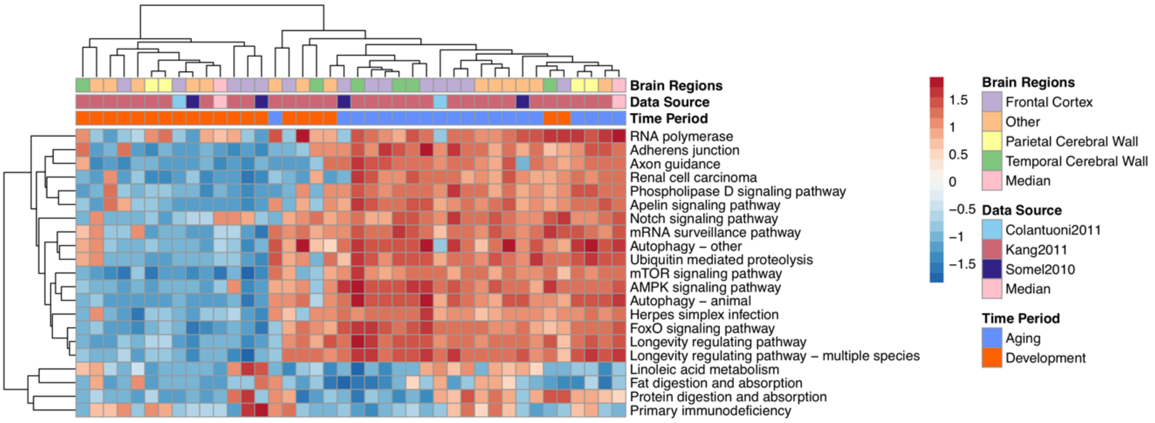

Figure S15: A heatmap of the normalized enrichment scores for KEGG pathways that are significantly associated with consistent heterogeneity in aging (rows) and datasets (columns). Datasets also include a new column, named Median, which includes, for each gene, the median of the changes in heterogeneity across datasets. The color represents normalized enrichment scores; red: positive, blue: negative and the darkness specifies how strong the association is.

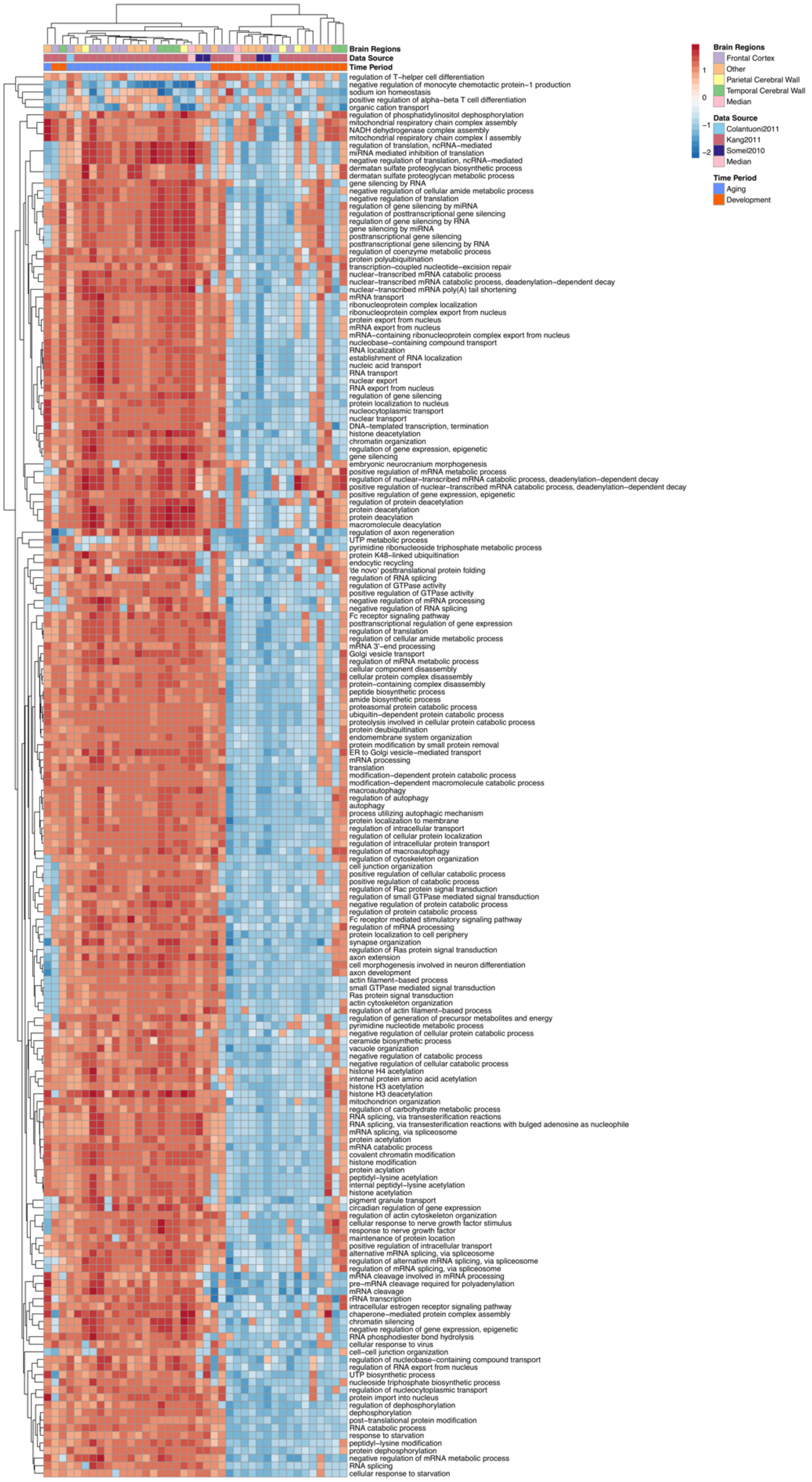

Figure S16: The same as Figure S15 but for Gene Ontology Biological Process categories.

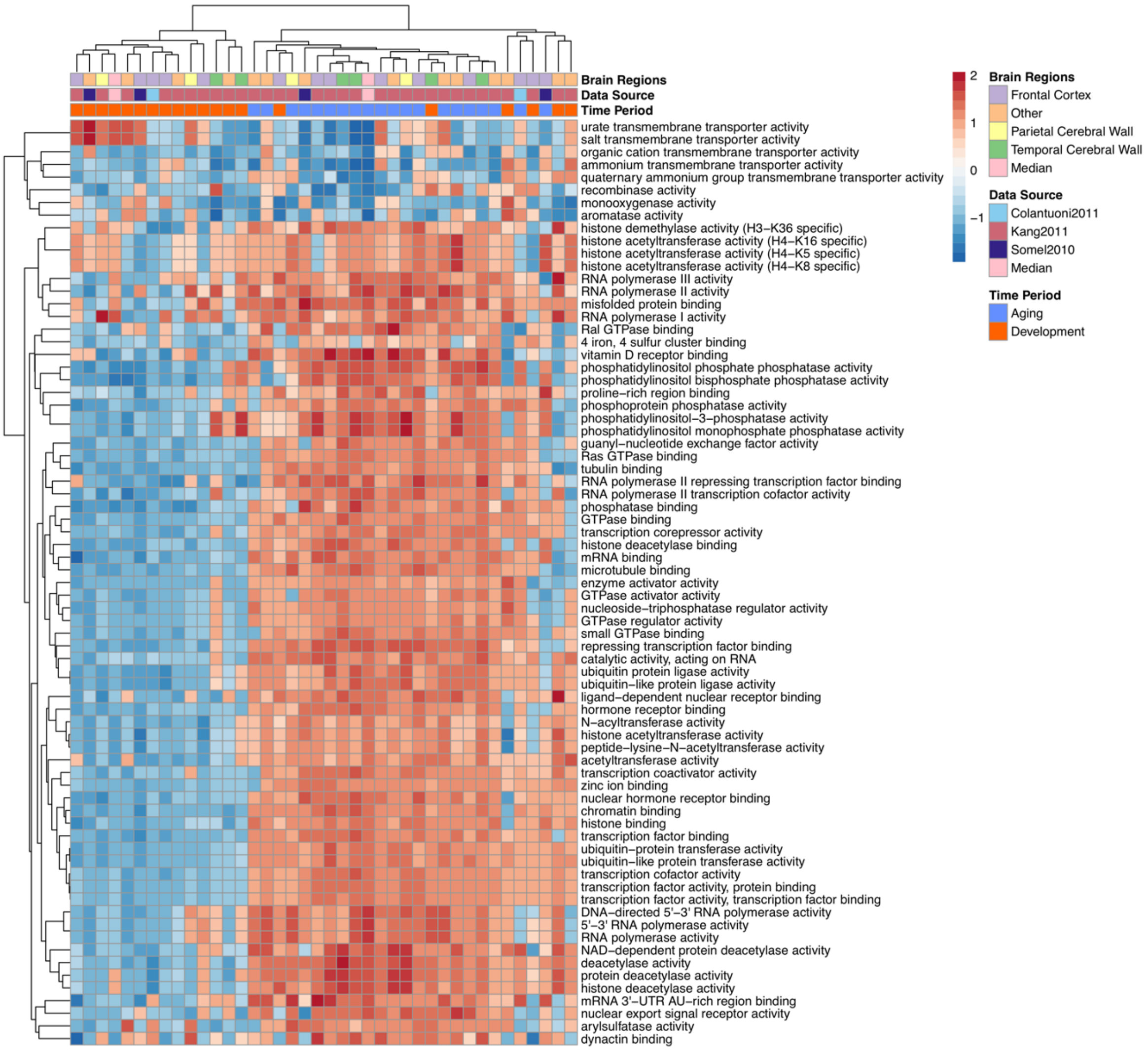

Figure S17: The same as Figure S15 but for Gene Ontology Molecular Function categories.

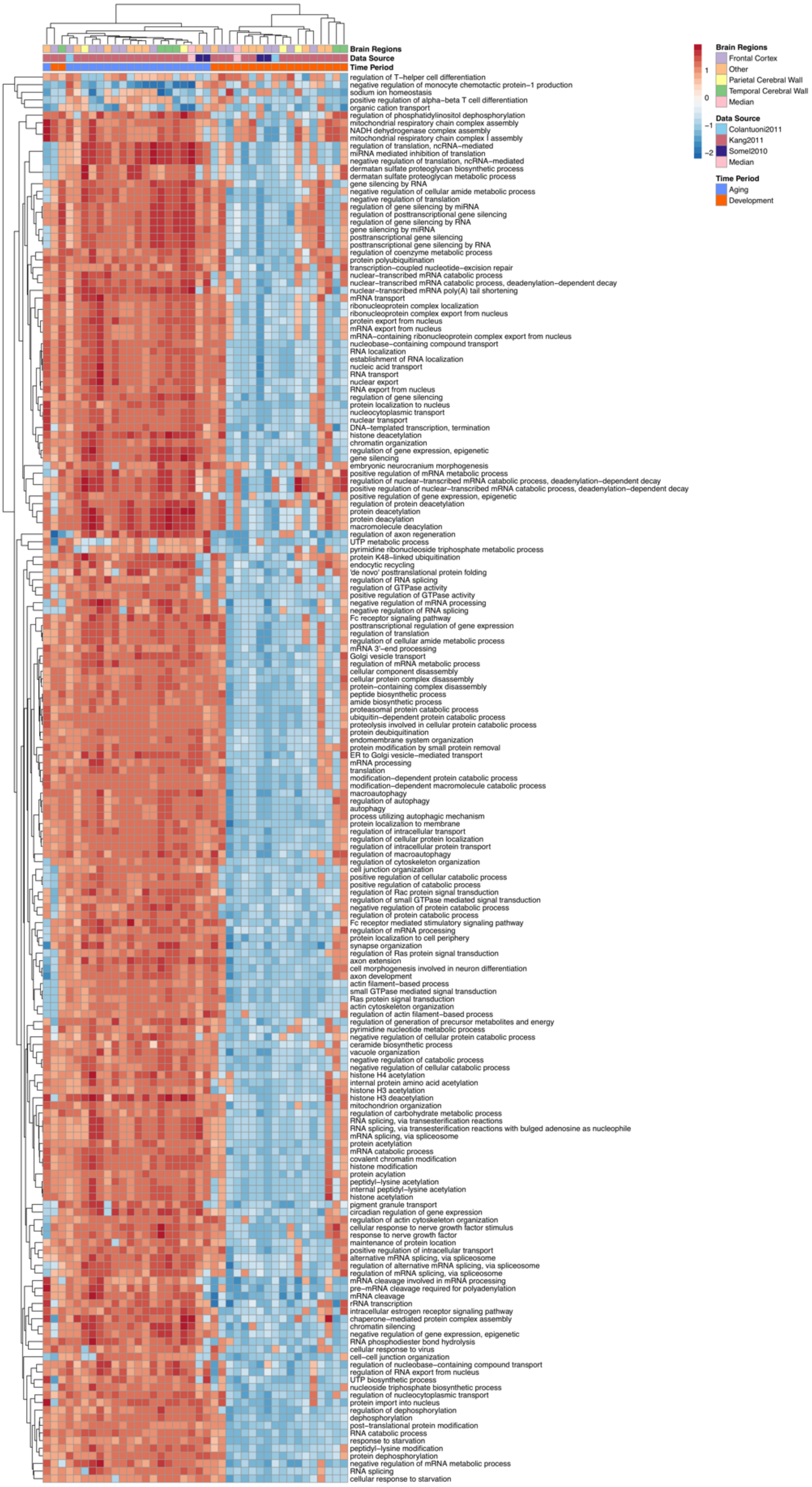

Figure S18: The same as Figure S15 but for Gene Ontology Cellular Component categories.

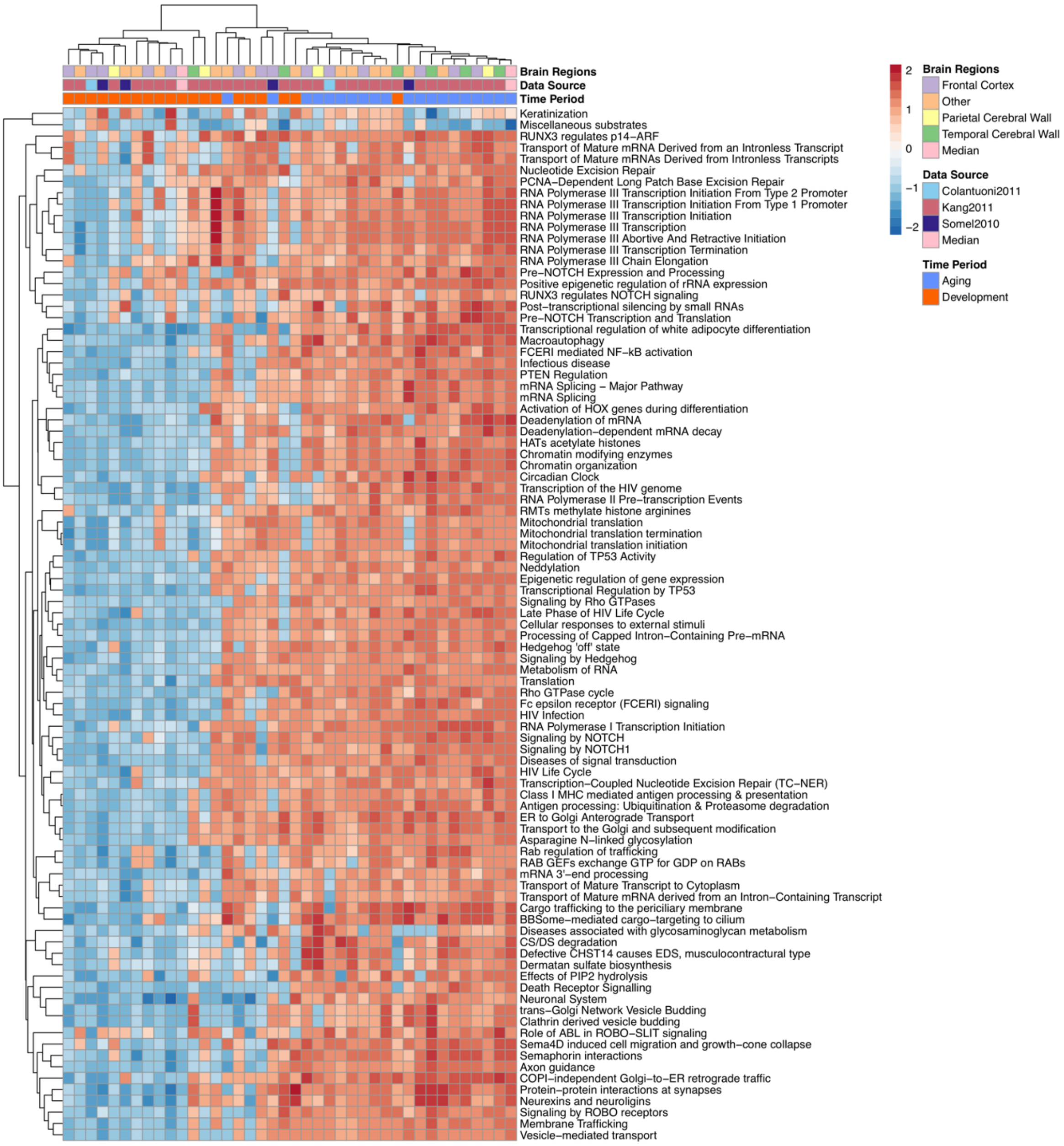

Figure S19: The same as Figure S15 but for Reactome pathways.

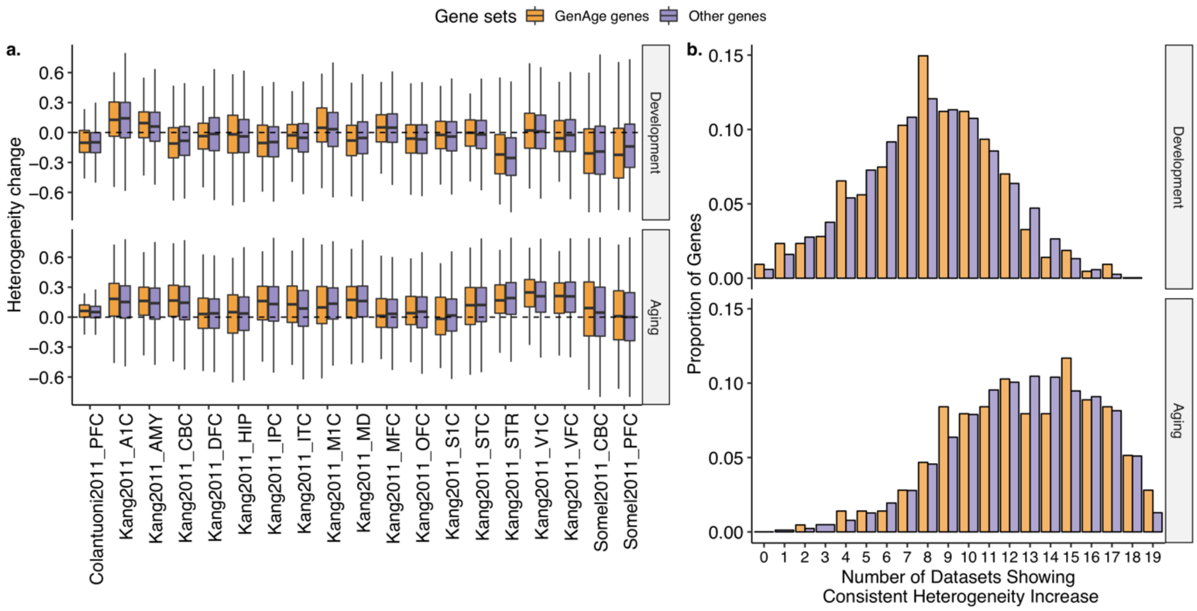

Figure S20: Association between GenAge human gene set (*n* = 214) and age-related heterogeneity. (a) Boxplots show age-related heterogeneity changes (ρ values) (y-axis) of genes from GenAge gene set (orange) and the remaining genes (purple) in different datasets (x-axis) during aging (lower panel) and development (upper panel). (b) Consistency in age-related heterogeneity increase in genes from GenAge gene set (orange) and the other genes (purple) in aging (lower panel) and development (upper panel). The x-axis shows the number of datasets among which genes show consistent heterogeneity increase, while the y-axis shows the proportions of the number of genes.

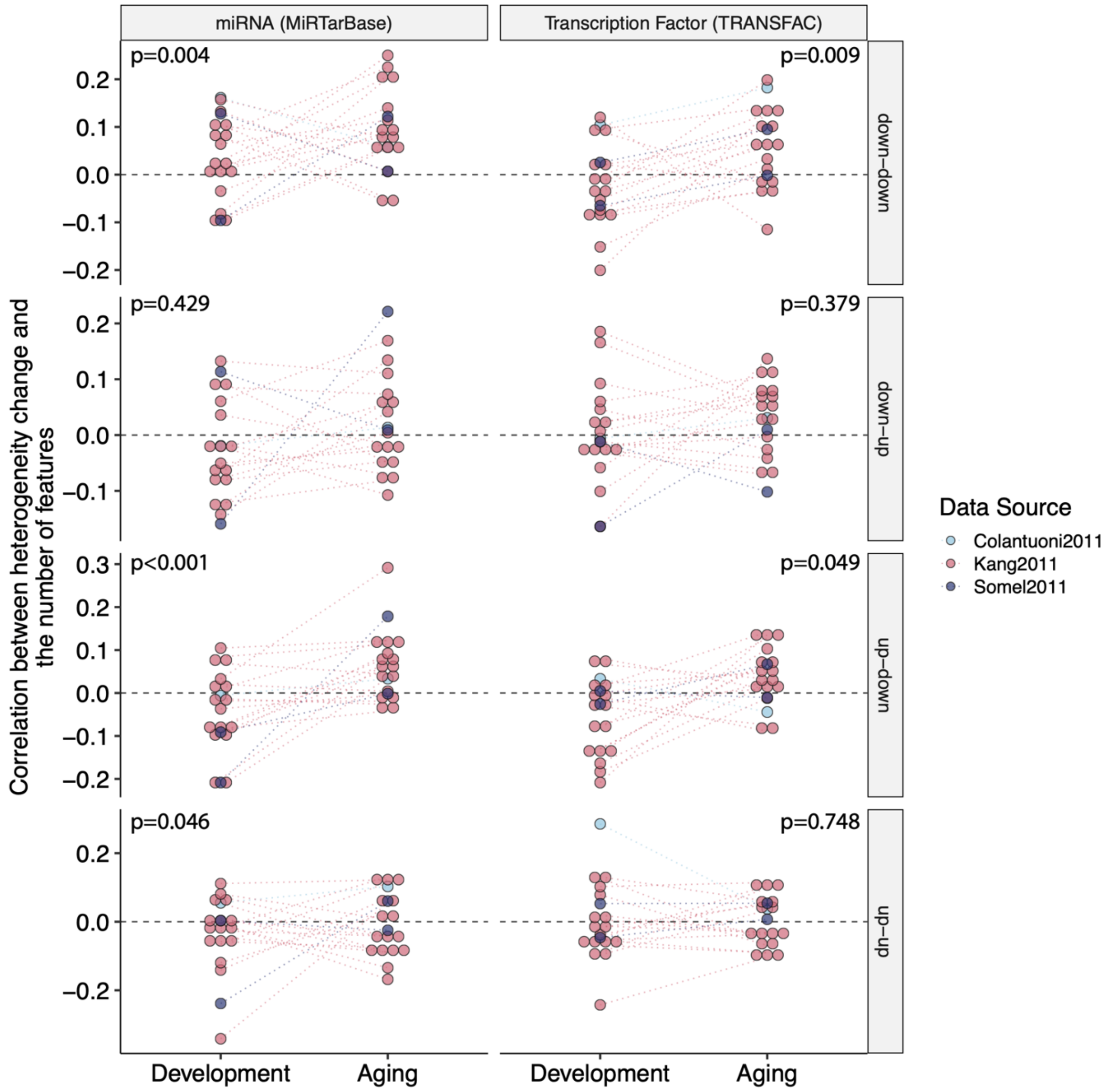

Figure S21: Correlation between the change in heterogeneity and number of transcriptional regulators, i.e. miRNA and transcription factors. Each point represents a dataset, and the color shows the data source. *p*-values are calculated using a permutation test. The dashed line at y = 0 shows zero correlation. Genes were divided into four sets based on the change in their expression level in development and aging, e.g. “down-down” includes genes with decreased expression in both development and aging, whereas “down-up” includes genes with decreasing expression level in development and then increase in aging.

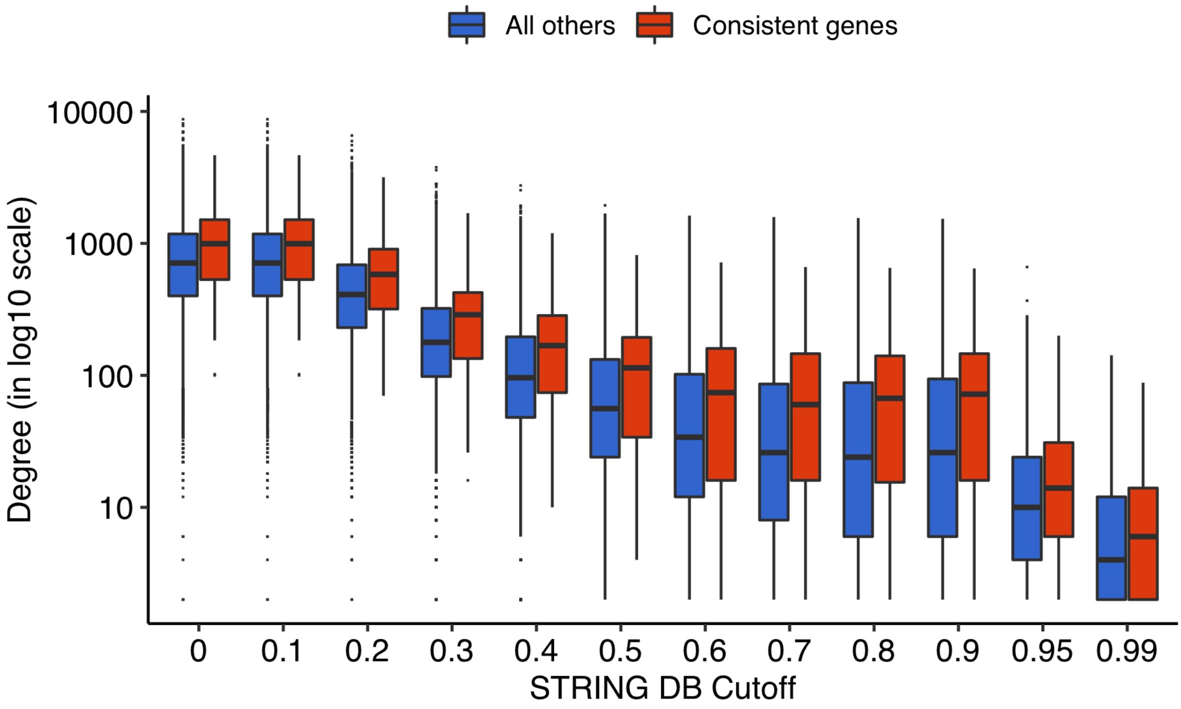

Cutoff	Number of Genes	Number of Interactions	p	FDR
0.00	11,016	4,920,316	<0.0001	<0.0001
0.10	11,016	4,920,316	<0.0001	<0.0001
0.20	11,015	2,918,350	0.0001	0.0001
0.30	11,015	1,376,452	<0.0001	<0.0001
0.40	11,008	845,298	<0.0001	<0.0001
0.50	10,955	578,970	<0.0001	<0.0001
0.60	10,665	438,686	<0.0001	<0.0001
0.70	9,881	353,362	<0.0001	<0.0001
0.80	8,588	305,128	<0.0001	<0.0001
0.90	7,386	270,942	0.0001	0.0001
0.95	5,800	58,190	0.0235	0.0256
0.99	3,167	17,660	0.3989	0.3989

Figure S22: Degree distributions in protein-protein interaction database (STRING) for consistent genes (red) and all others (blue). The y-axis show degree (number of interactors) in log scale. The x-axis shows different cutoffs for interaction confidence used to filter STRING database. The color represents two sets of genes; red: consistent genes that show consistent increase in heterogeneity across all aging datasets, blue: all other genes. Interaction degree is significantly higher in consistent genes across all cutoffs except for 0.99 (permutation test). Details, including the number of genes, interactions and *p*-values, are given as a table.

Cell Type	Odds Ratio	FDR	Total Cell–type	Total Heterogeneus	a	b	c	d	p
OLs	5.537	0.179	51	9	2	49	7	953	0.072
Myelin_OLs	3.854	0.179	348	9	6	342	3	660	0.071
OPCs	0.265	0.357	322	9	1	321	8	681	0.286
Neurons	0.000	0.205	272	9	0	272	9	730	0.123
Astrocytes	0.000	1.000	18	9	0	18	9	984	1.000

Figure S23: Cell-type specificity analysis for genes that become heterogenous with age across all aging datasets. Fisher's exact test is used to calculate the association (Odds Ratio) and *p*-value. FDR corrected *p*-value (FDR column) is also given. "Total Cell-type" column is the total number of cell-type-specific genes, "Total Heterogeneous" is the total number of genes in the 147 consistently heterogeneous genes and are cell-type specific for at least one cell type, "a" is the overlap between cell-type specific genes and heterogeneous for a given cell-type, "b" is the cell-type specific genes that are not among the 147 genes but in the dataset, "c" is the number of heterogeneous genes that are specific for other cell-types, and "d" is the total number of genes that are specific for at least one cell-type and measured in the age-series datasets.

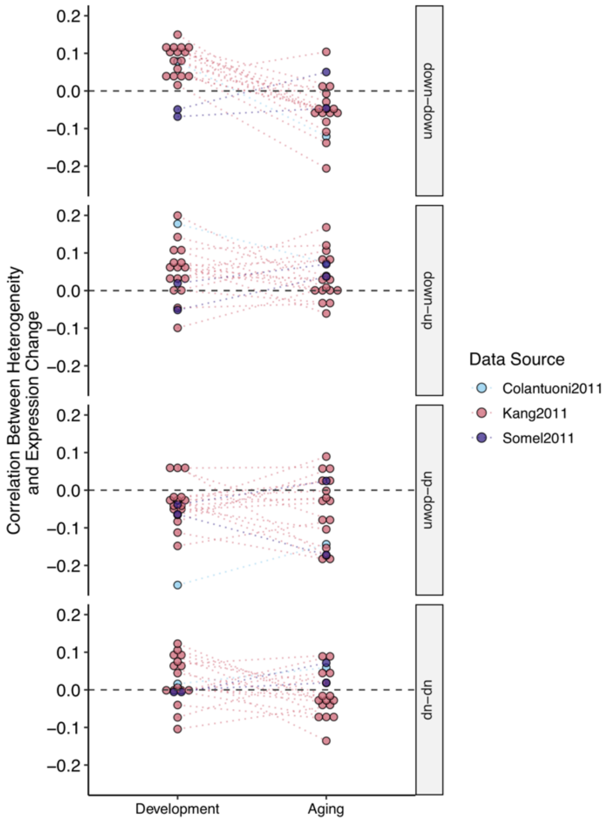

Figure S24: The relationship between the age-related changes in gene expression (β values) and changes in heterogeneity (ρ values). The y-axis shows the Spearman's correlation coefficients, while different periods are in the x-axis. Facets show different trends in expression changes: up-down: up-regulation in development and down-regulation in aging; down-up: down-regulation in development and up-regulation in aging; up-up: up-regulation in development and up-regulation in aging; down-down: down-regulation in development and down-regulation in aging.

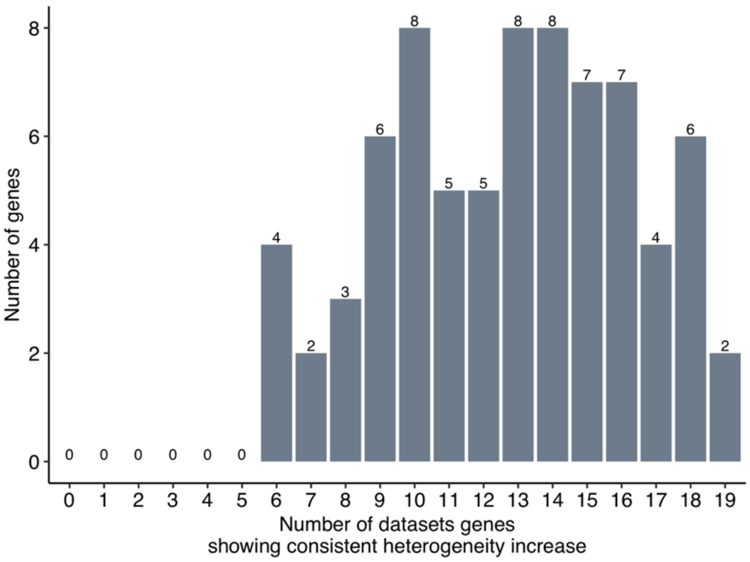

Figure S25: Analysis of the consistency in aging-related heterogeneity of the post-mortem interval (PMI)-associated genes. The x-axis shows the number of datasets in which genes were showing a consistent increase in age-related heterogeneity during aging, while y-axis reflects the number of genes. There were 105 previously identified PMI associated genes in the human cerebral cortex (see main text), 75 of which were included in our analyses. We asked if these PMI associated genes show more increase in heterogeneity, and found that only 2 of 147 consistent genes (i.e. genes showing an increase in heterogeneity in 19 out of 19 aging datasets) were associated with PMI.

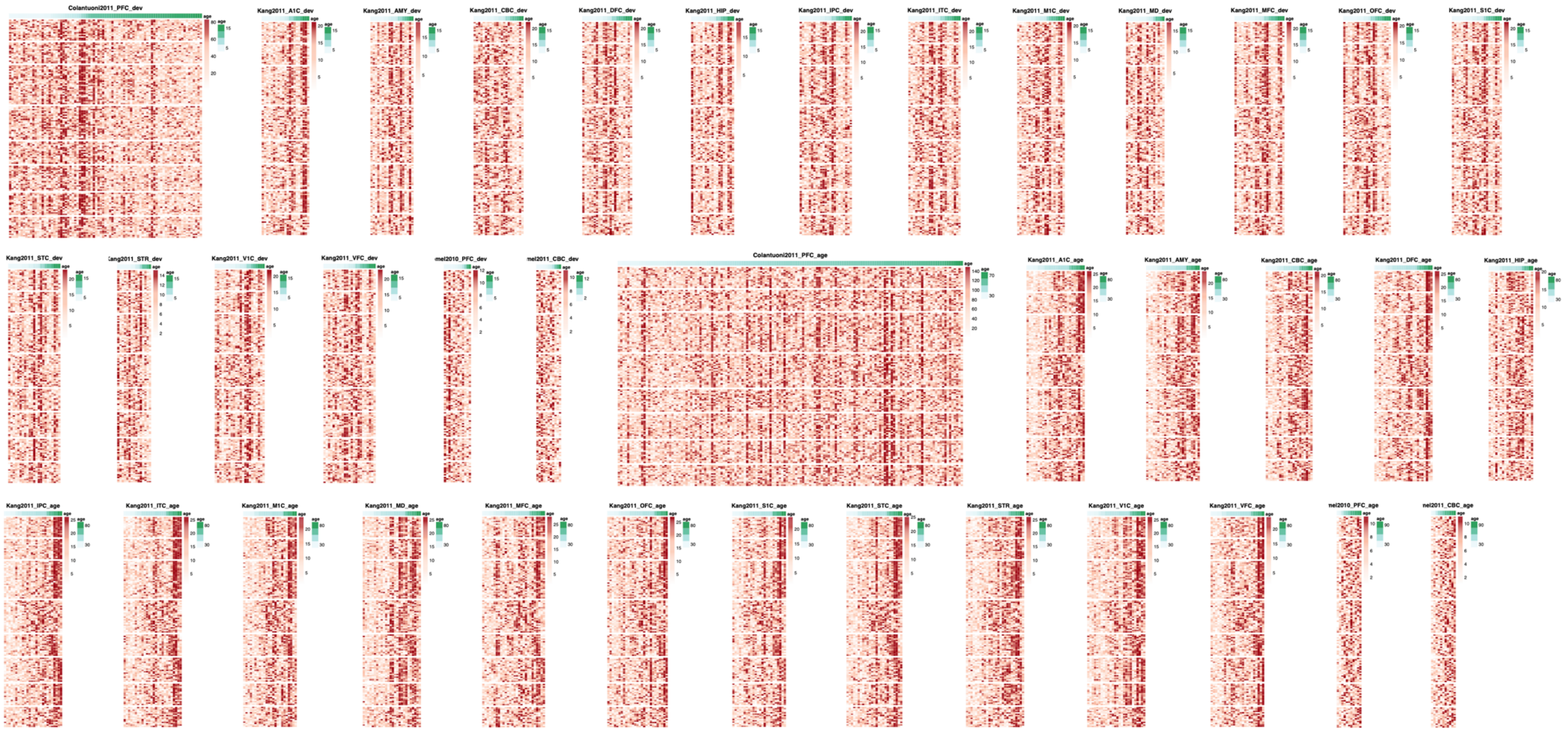

Figure S26: Heatmaps for 38 datasets showing the heterogeneity levels (residuals) for each individual (columns) in 147 genes that show consistent increase across all aging datasets (rows). The color shows the rank of individuals with respect to their heterogeneity for that particular gene. Darker colors show individuals with the highest heterogeneity for that particular gene. Genes are clustered using the heterogeneity trajectories in Figure 4 and individuals are ordered by age (darker green in column annotation showing older ages).

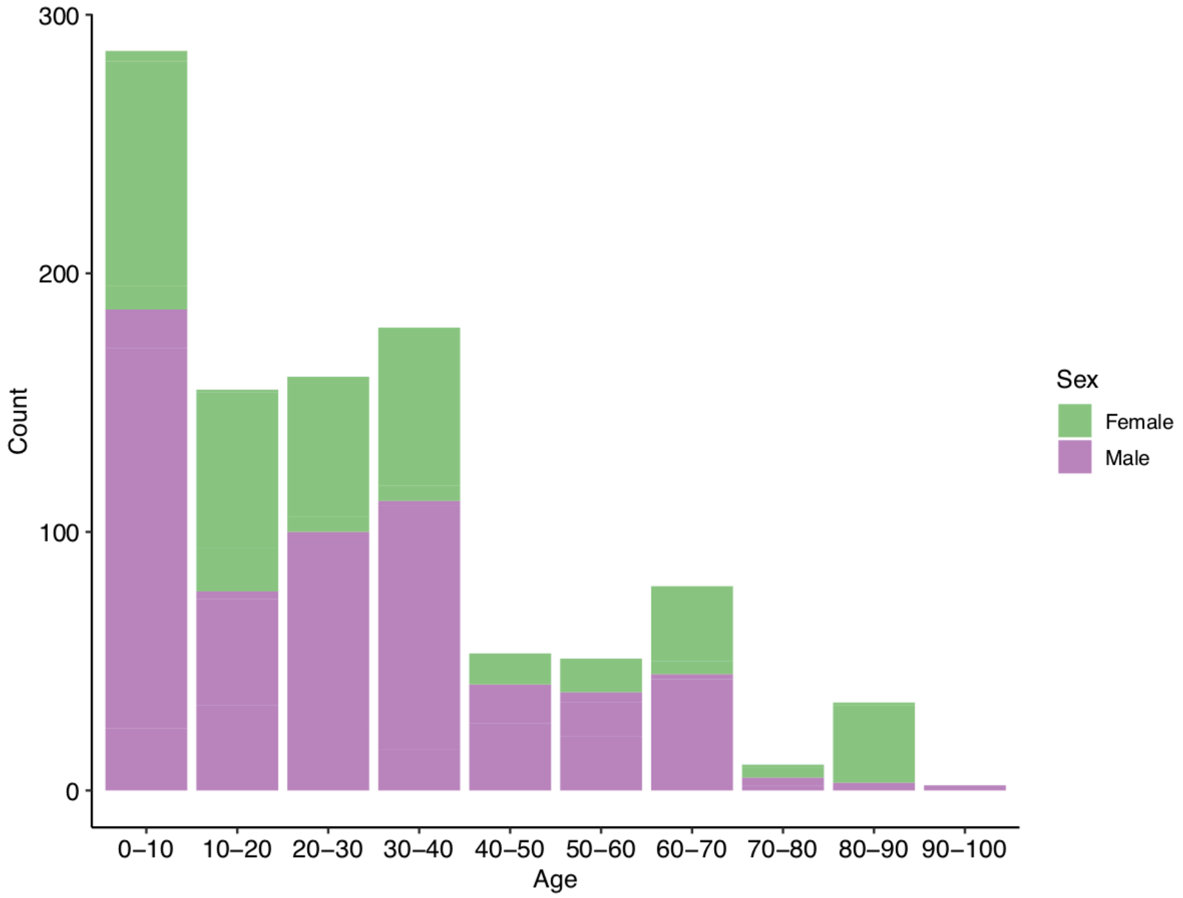

Figure S27: The proportions of sexes of samples used in the analysis. The x- and y-axes show age intervals and number of samples, respectively. The colors specify the proportion of male (purple) and female (green) samples.

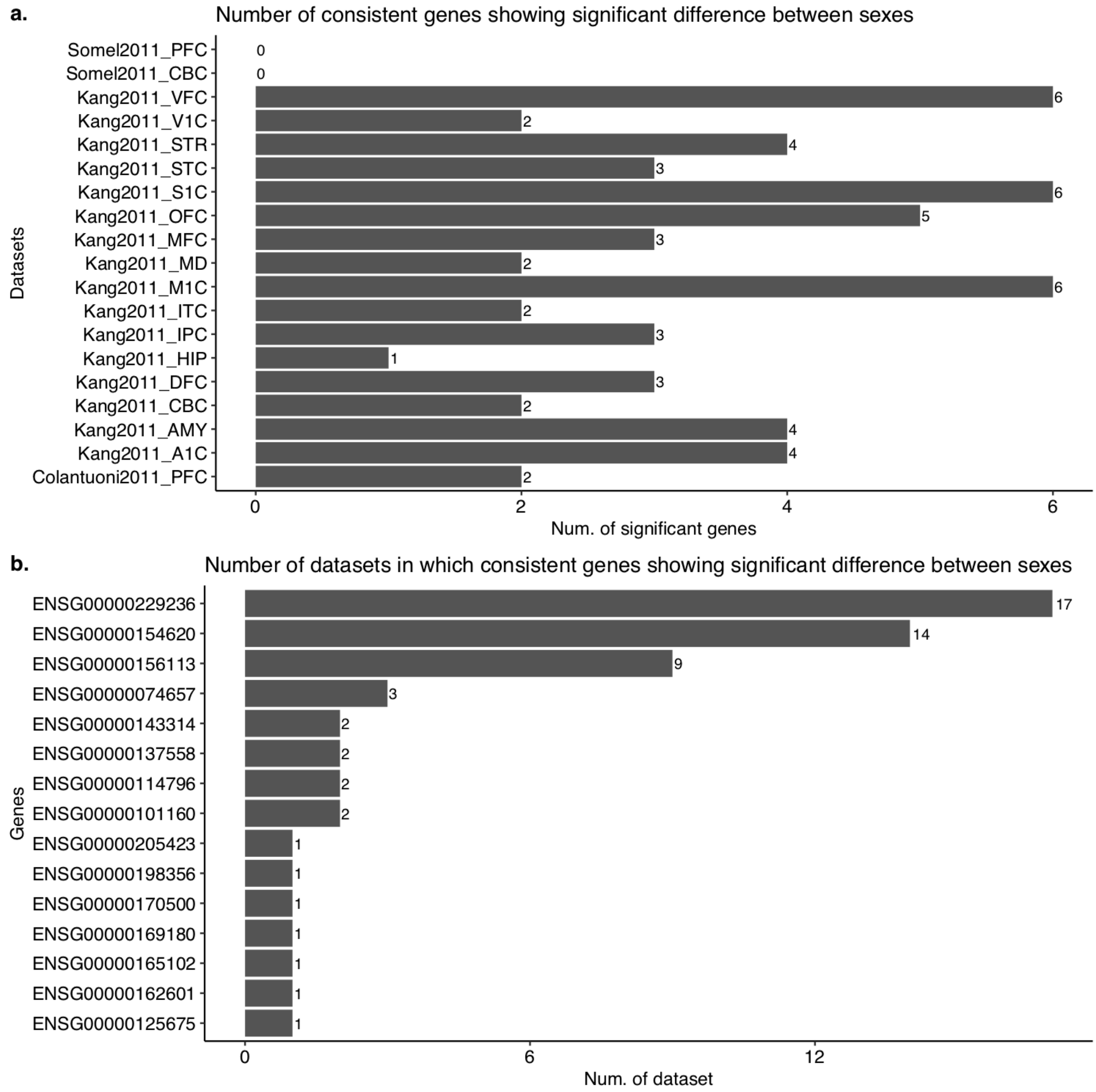

Figure S28: Genes showing significant difference (FDR corrected $p < 0.05$) in expression trajectories between males and females. Two sample Wilcoxon test was performed using residual values obtained from equation (1) (see Methods) to determine if the distribution of residuals differs between different sexes. (a) Number of genes showing significant difference in each dataset. (b) Number of datasets in which the genes show significant difference. In total, 15/147 genes show significant change in at least one dataset.

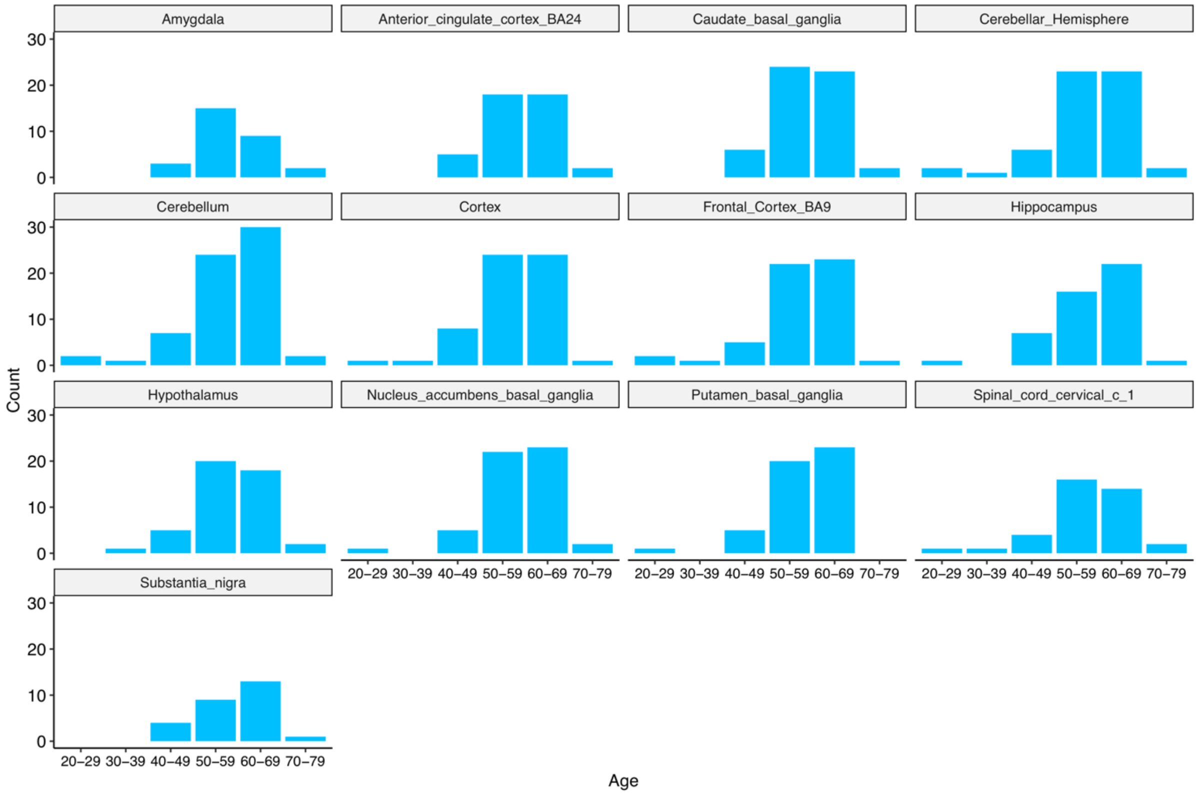

Figure S29: Age distribution of the individuals used in GTEx RNA-Seq datasets.

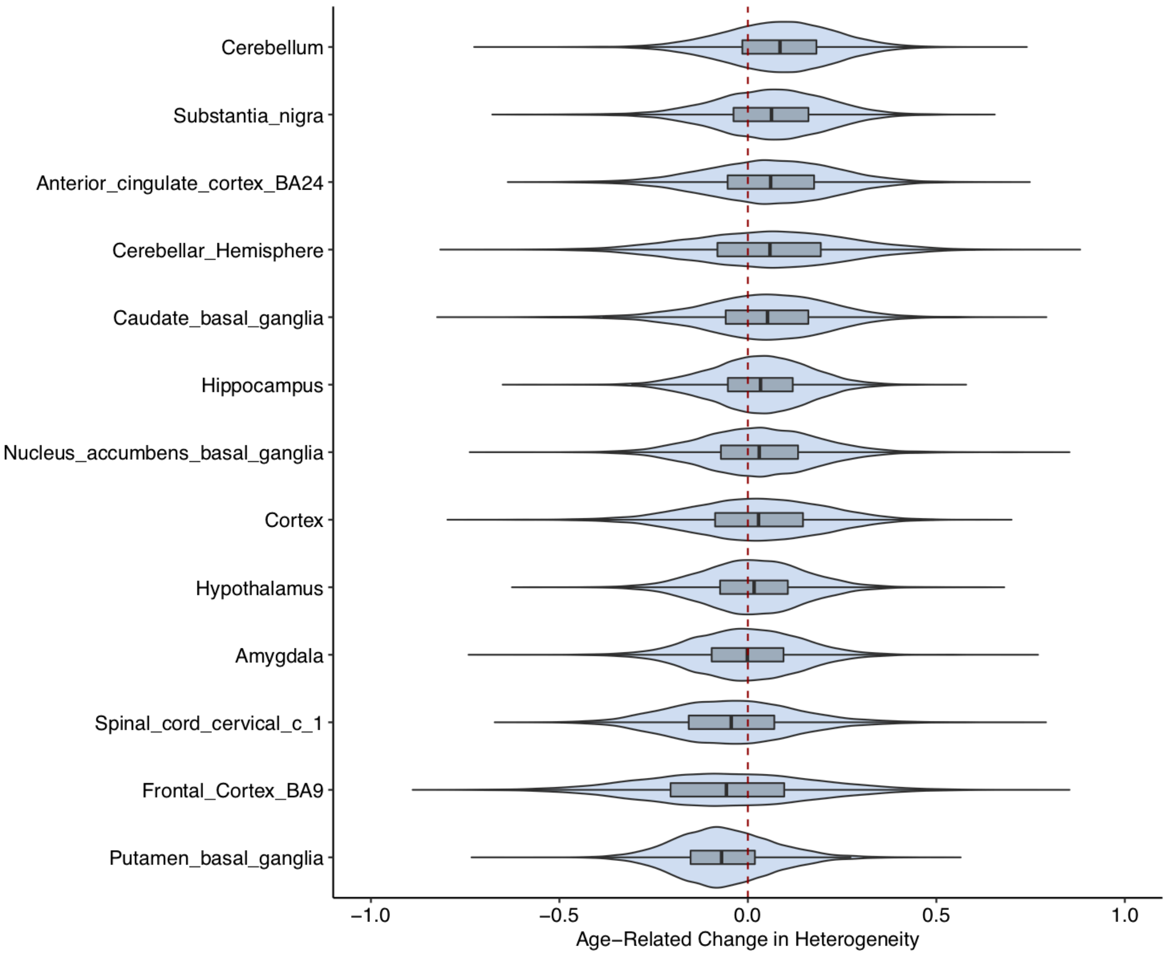

Figure S30: Distribution of age-related changes in heterogeneity (rho values) (x-axis) in different GTEx datasets, corresponding to different brain regions in (y-axis). The red dotted vertical line (at x = 0) reflects no change in heterogeneity.

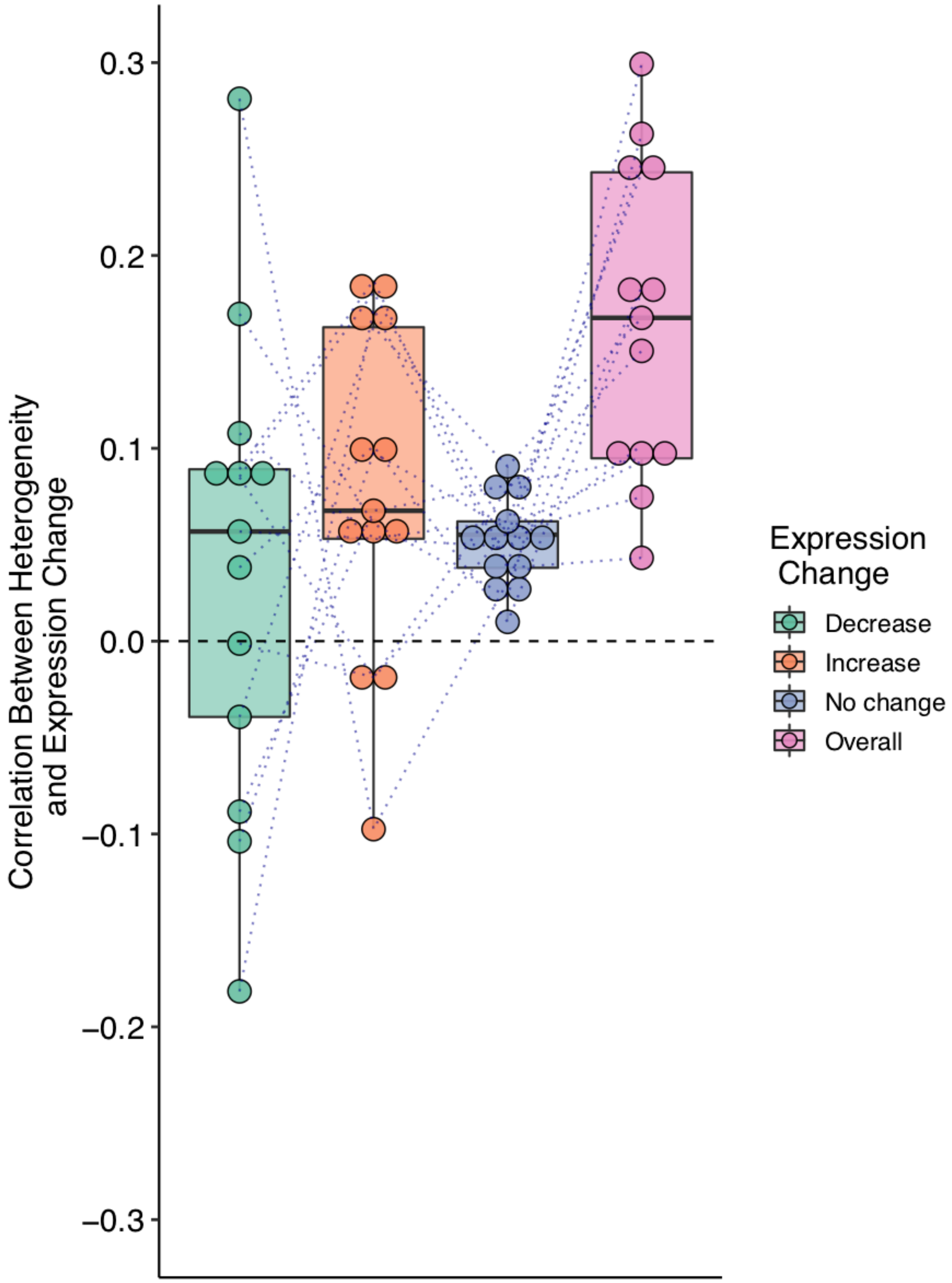

Figure S31: The relationship between expression and heterogeneity changes in GTEx datasets. The y-axis shows Spearmen correlation coefficients calculated between age-related expression changes (β values) and heterogeneity changes (ρ values) separately for each dataset. Different colors indicate the direction of expression change. Genes with the beta values within the range of -0.1 and 0.1 were assumed to be no change. The overall includes all the genes irrespective of the direction of their expression change.

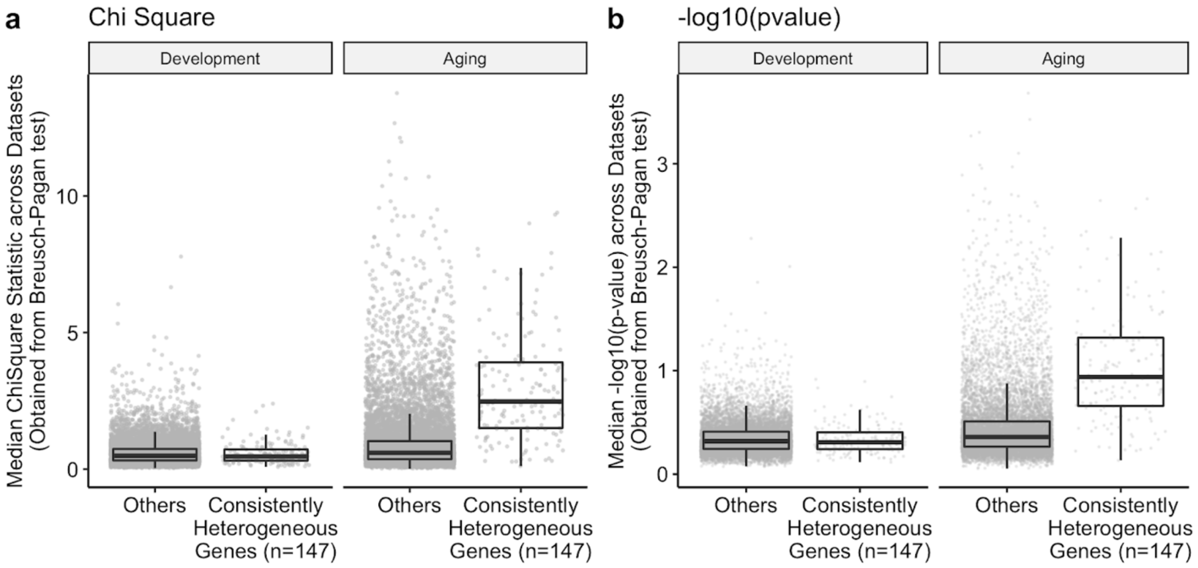

Figure S32: Distribution of a) median Chi Square values and b) median -log10 p values for each gene obtained from Breusch-Pagan heteroskedasticity test, stratified for the changes in development and aging. The 147 genes that become heterogeneous with age consistently in aging datasets shown separately to compare with other genes.

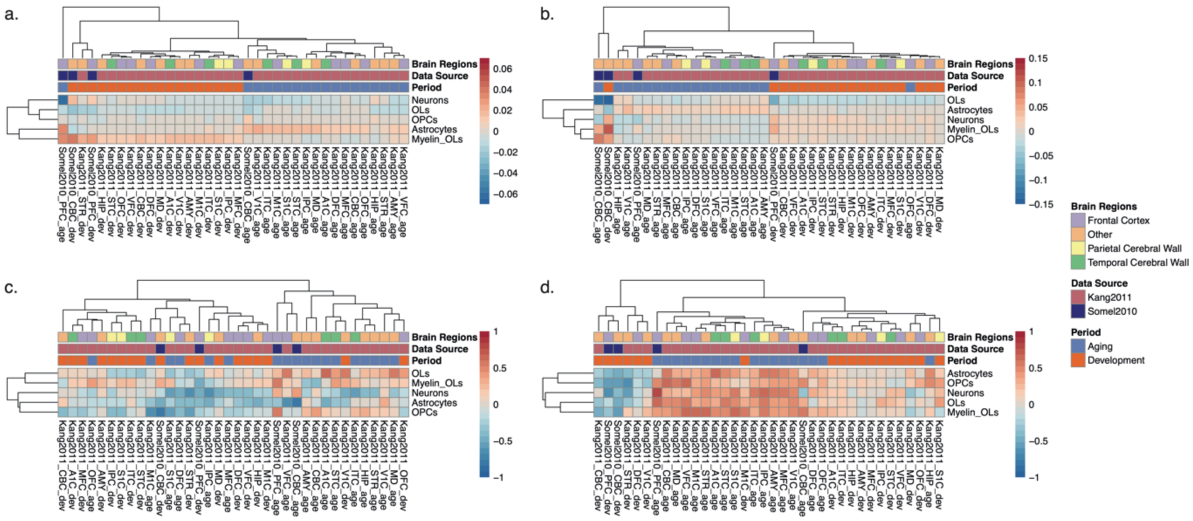

Figure S33: a) The change in cell-type proportions with age in different datasets (beta values from a linear model between cell-type proportions and age in fourth root scale), b) The change in cell-type proportions based on the 147 genes that become more heterogeneous with age during aging, c) The change in cell-type heterogeneity with age (correlation between the absolute value of residuals from the linear model and age), d) The change in cell-type heterogeneity with age, calculated only using the 147 genes.

All Pathways

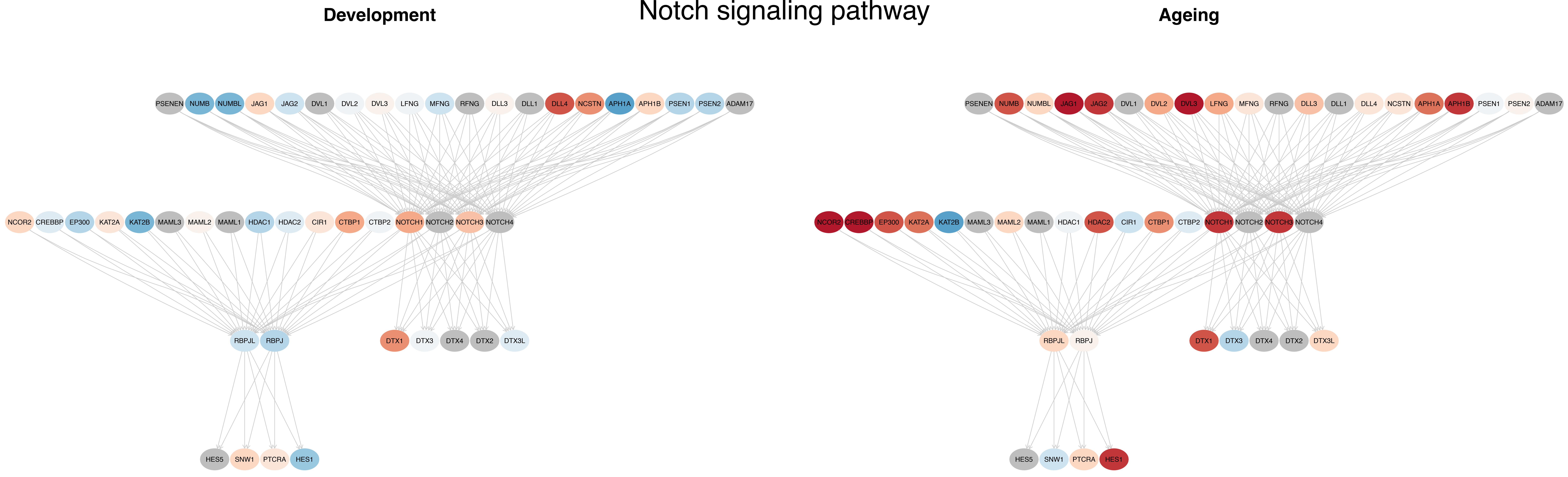
Notch signaling pathway
Development
PSENEN NUMB NUMBL JAG1 JAG2 DVL1 DVL2 DVL3 LFNG MFNG RFNG DLL3 DLL1 DLL4 NCSTN APH1A APH1B PSEN1 PSEN2 ADAM17
NCOR2 CREBBP EP300 KAT2A KAT2B MAML3 MAML2 MAML1 HDAC1 HDAC2 CIR1 CTBP1 CTBP2 NOTCH1 NOTCH2 NOTCH3 NOTCH4
RBPJL RBPJ
DTX1 DTX3 DTX4 DTX2 DTX3L
HES5 SNW1 PTCRA HES1
Ageing
PSENEN NUMB NUMBL JAG1 JAG2 DVL1 DVL2 DVL3 LFNG MFNG RFNG DLL3 DLL1 DLL4 NCSTN APH1A APH1B PSEN1 PSEN2 ADAM17
NCOR2 CREBBP EP300 KAT2A KAT2B MAML3 MAML2 MAML1 HDAC1 HDAC2 CIR1 CTBP1 CTBP2 NOTCH1 NOTCH2 NOTCH3 NOTCH4
RBPJL RBPJ
DTX1 DTX3 DTX4 DTX2 DTX3L
HES5 SNW1 PTCRA HES1

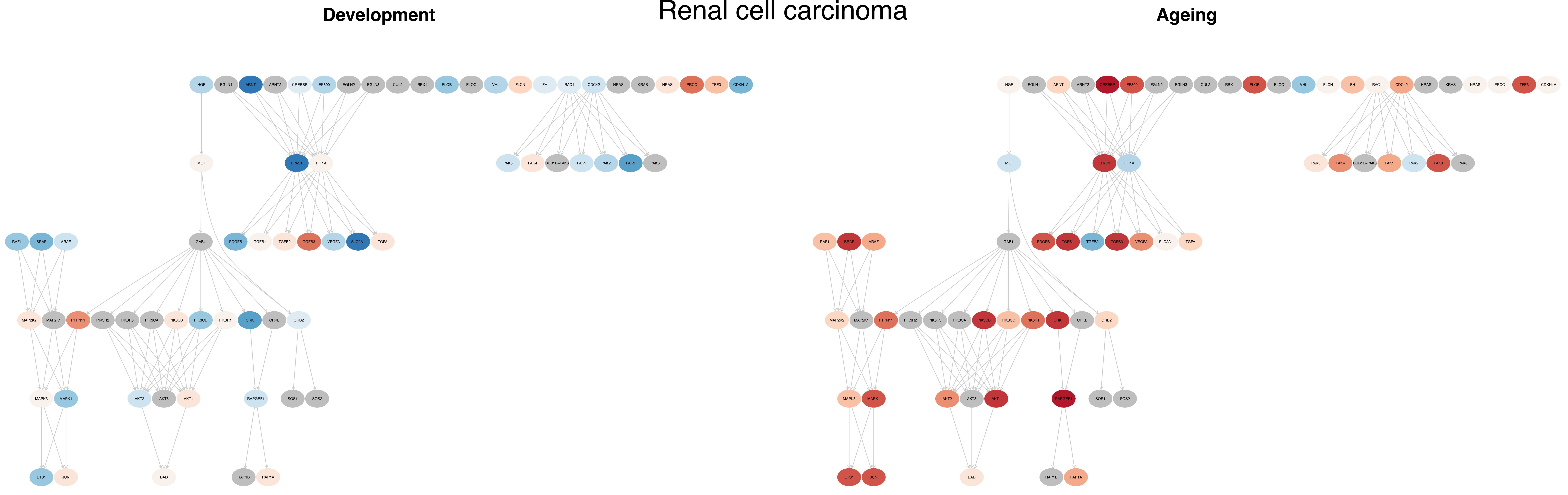
Renal cell carcinoma
Development
Ageing
HGF EGLN1 ARNT ARNT2 CREBBP EP300 EGLN2 EGLN3 CUL2 RBX1 ELOB ELOC VHL FLCN FH RAC1 CDC42 HRAS KRAS NRAS PRCC TFE3 CDKN1A
MET EPAS1 HIF1A PAK5 PAK4 BUB1B-PAK6 PAK1 PAK2 PAK3 PAK6
RAF1 BRAF ARAF GAB1 PDGFB TGFB1 TGFB2 TGFB3 VEGFA SLC2A1 TGFA
MAP2K2 MAP2K1 PTPN11 PIK3R2 PIK3R3 PIK3CA PIK3CB PIK3CD PIK3R1 CRK CRKL GRB2
MAPK3 MAPK1 AKT2 AKT3 AKT1 RAPGEF1 SOS1 SOS2
ETS1 JUN BAD RAP1B RAP1A

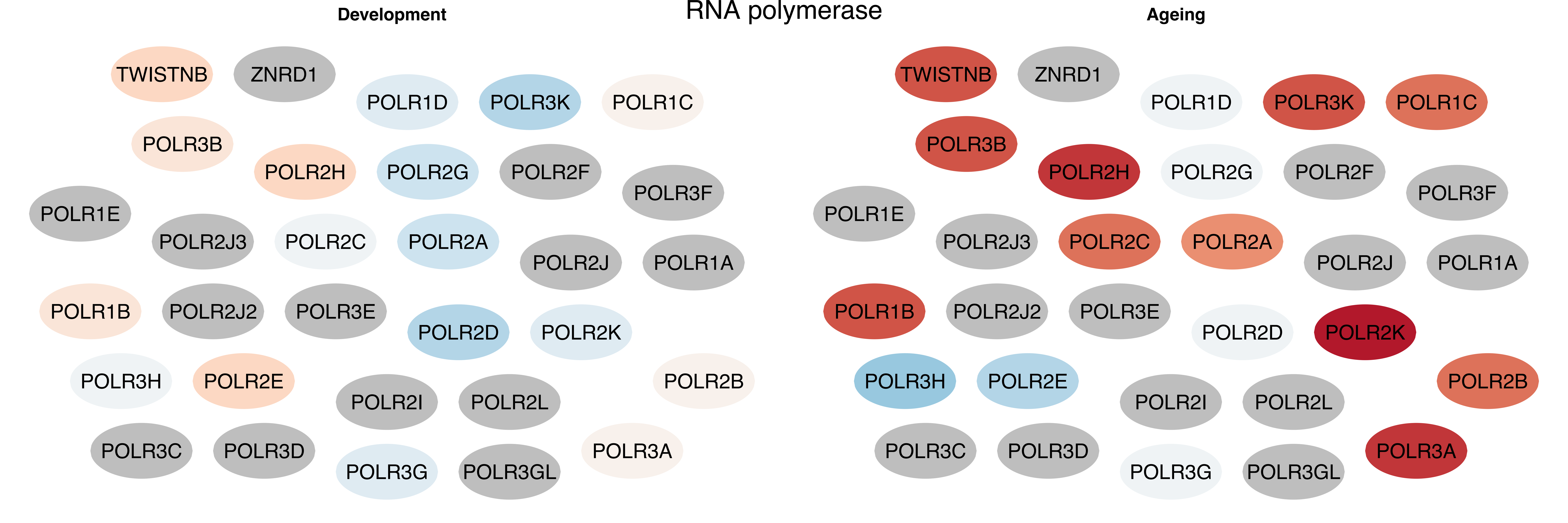
RNA polymerase
Development
TWISTNB
ZNRD1
POLR1D
POLR3K
POLR1C
POLR3B
POLR2H
POLR2G
POLR2F
POLR3F
POLR1E
POLR2J3
POLR2C
POLR2A
POLR2J
POLR1A
POLR1B
POLR2J2
POLR3E
POLR2D
POLR2K
POLR3H
POLR2E
POLR2I
POLR2L
POLR2B
POLR3C
POLR3D
POLR3G
POLR3GL
POLR3A
Ageing
TWISTNB
ZNRD1
POLR1D
POLR3K
POLR1C
POLR3B
POLR2H
POLR2G
POLR2F
POLR3F
POLR1E
POLR2J3
POLR2C
POLR2A
POLR2J
POLR1A
POLR1B
POLR2J2
POLR3E
POLR2D
POLR2K
POLR3H
POLR2E
POLR2I
POLR2L
POLR2B
POLR3C
POLR3D
POLR3G
POLR3GL
POLR3A

mRNA surveillance pathway

Development **Ageing**

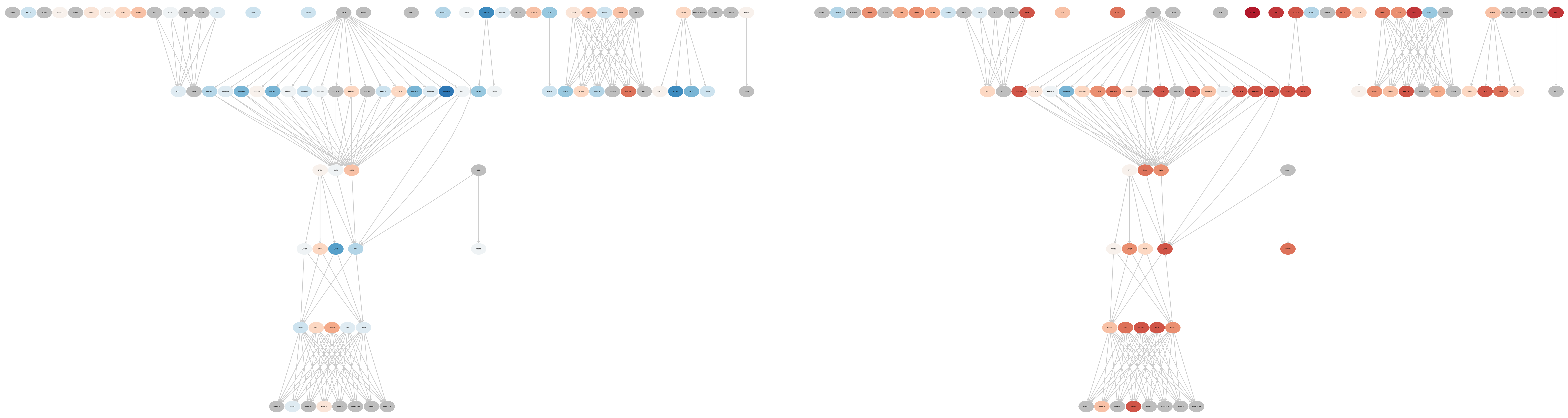

Autophagy – other

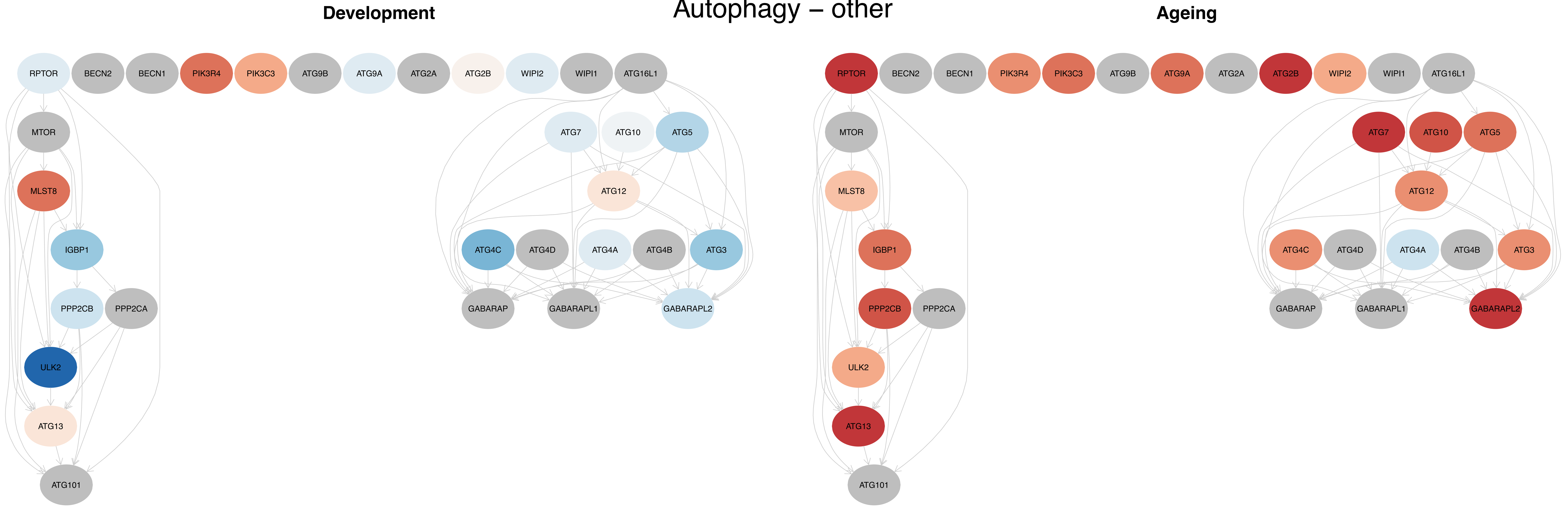

AMPK signaling pathway

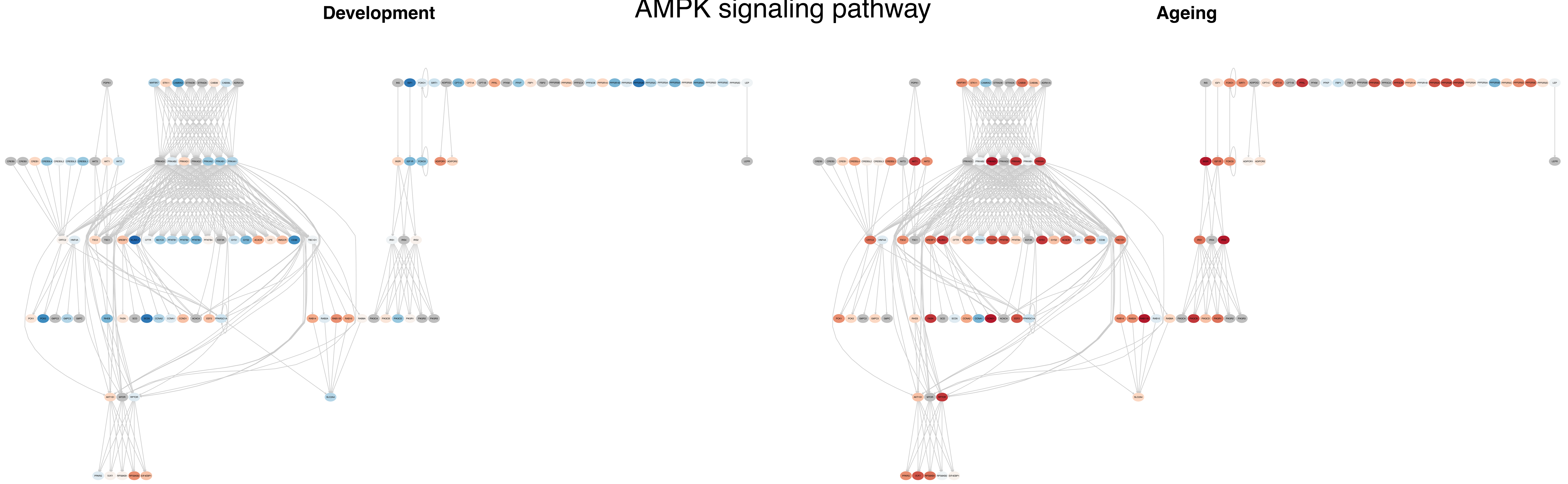

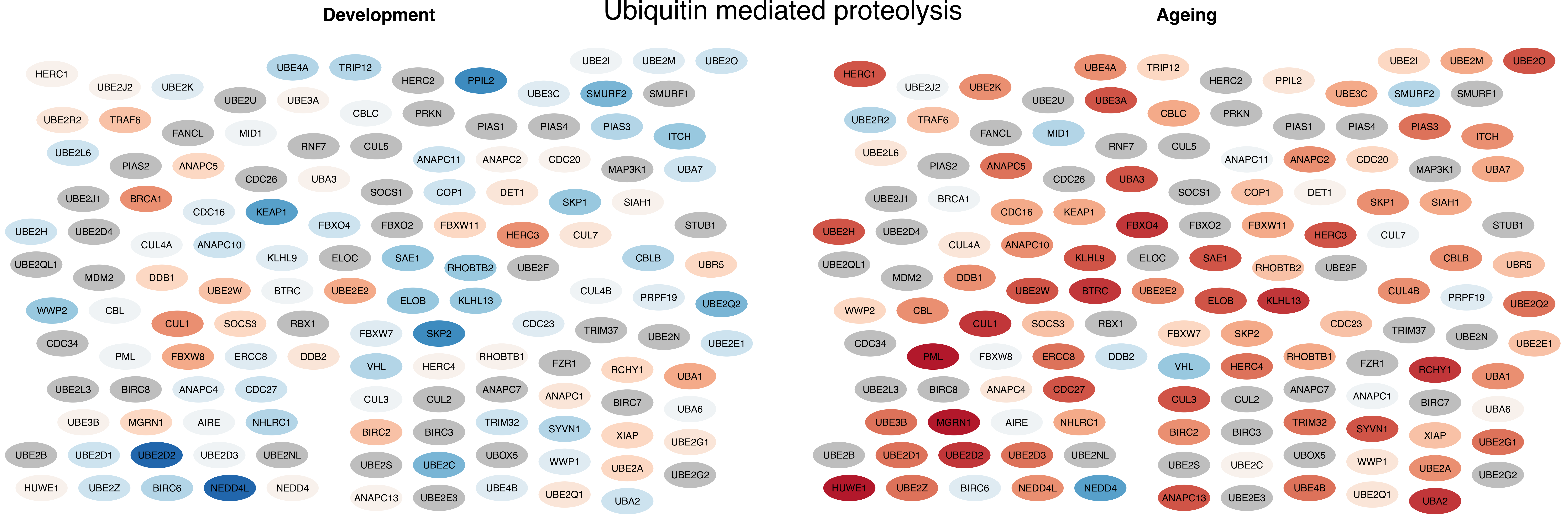
Ubiquitin mediated proteolysis
Development
HERC1 UBE2J2 UBE2K UBE2U UBE3A UBE4A TRIP12 HERC2 PPIL2 UBE2I UBE2M UBE2O UBE3C SMURF2 SMURF1 CBLC PRKN UBE2R2 TRAF6 FANCL MID1 PIAS1 PIAS4 PIAS3 ITCH RNF7 CUL5 UBE2L6 ANAPC11 ANAPC2 CDC20 PIAS2 ANAPC5 MAP3K1 UBA7 CDC26 UBA3 SOCS1 COP1 DET1 UBE2J1 BRCA1 SKP1 SIAH1 CDC16 KEAP1 FBXO4 FBXO2 FBXW11 STUB1 UBE2H UBE2D4 HERC3 CUL7 CUL4A ANAPC10 KLHL9 ELOC SAE1 RHOBTB2 UBE2F CBLB UBE2QL1 UBR5 MDM2 DDB1 UBE2W BTRC UBE2E2 CUL4B ELOB KLHL13 PRPF19 UBE2Q2 WWP2 CBL CUL1 SOCS3 RBX1 CDC23 TRIM37 FBXW7 SKP2 UBE2N UBE2E1 CDC34 PML FBXW8 ERCC8 DDB2 RHOBTB1 VHL HERC4 FZR1 RCHY1 UBA1 UBE2L3 BIRC8 ANAPC4 CDC27 ANAPC7 CUL3 CUL2 ANAPC1 BIRC7 UBA6 UBE3B MGRN1 AIRE NHLRC1 TRIM32 SYVN1 XIAP BIRC2 BIRC3 UBE2G1 UBE2B UBE2D1 UBE2D2 UBE2D3 UBE2NL UBOX5 WWP1 UBE2S UBE2C UBE2A UBE2G2 HUWE1 UBE2Z BIRC6 NEDD4L NEDD4 UBE4B UBE2Q1 ANAPC13 UBE2E3 UBA2
Ageing
HERC1 UBE2J2 UBE2K UBE2U UBE3A UBE4A TRIP12 HERC2 PPIL2 UBE2I UBE2M UBE2O UBE3C SMURF2 SMURF1 CBLC PRKN UBE2R2 TRAF6 FANCL MID1 PIAS1 PIAS4 PIAS3 ITCH RNF7 CUL5 UBE2L6 ANAPC11 ANAPC2 CDC20 PIAS2 ANAPC5 MAP3K1 UBA7 CDC26 UBA3 SOCS1 COP1 DET1 UBE2J1 BRCA1 SKP1 SIAH1 CDC16 KEAP1 FBXO4 FBXO2 FBXW11 STUB1 UBE2H UBE2D4 HERC3 CUL7 CUL4A ANAPC10 KLHL9 ELOC SAE1 RHOBTB2 UBE2F CBLB UBE2QL1 UBR5 MDM2 DDB1 UBE2W BTRC UBE2E2 CUL4B ELOB KLHL13 PRPF19 UBE2Q2 WWP2 CBL CUL1 SOCS3 RBX1 CDC23 TRIM37 FBXW7 SKP2 UBE2N UBE2E1 CDC34 PML FBXW8 ERCC8 DDB2 RHOBTB1 VHL HERC4 FZR1 RCHY1 UBA1 UBE2L3 BIRC8 ANAPC4 CDC27 ANAPC7 CUL3 CUL2 ANAPC1 BIRC7 UBA6 UBE3B MGRN1 AIRE NHLRC1 TRIM32 SYVN1 XIAP BIRC2 BIRC3 UBE2G1 UBE2B UBE2D1 UBE2D2 UBE2D3 UBE2NL UBOX5 WWP1 UBE2S UBE2C UBE2A UBE2G2 HUWE1 UBE2Z BIRC6 NEDD4L NEDD4 UBE4B UBE2Q1 ANAPC13 UBE2E3 UBA2

Phospholipase D signaling pathway

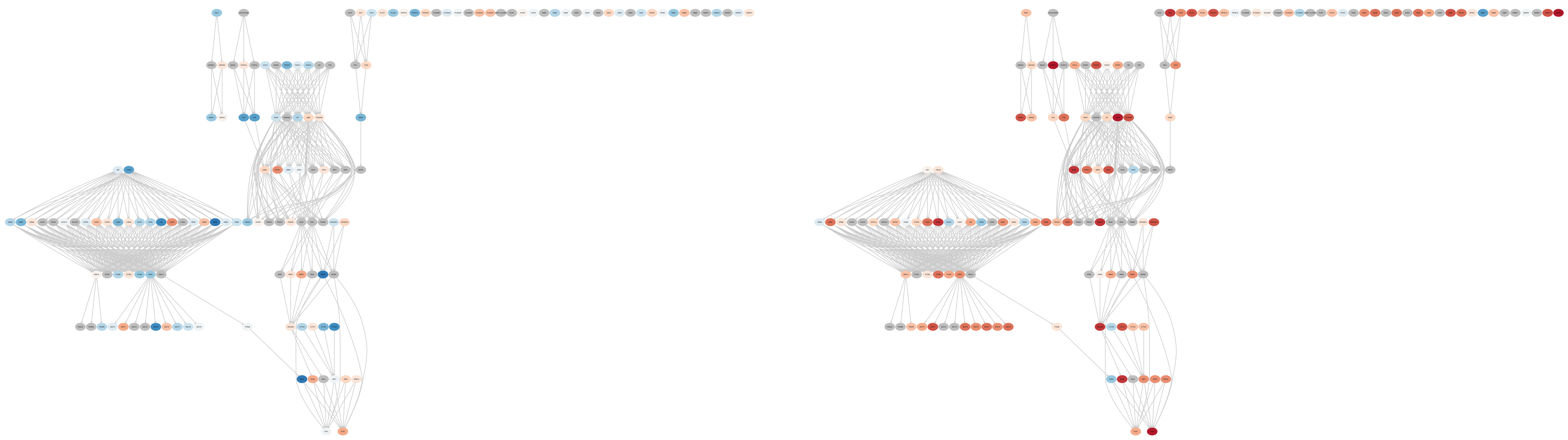

Longevity regulating pathway – multiple species

Development **Ageing**

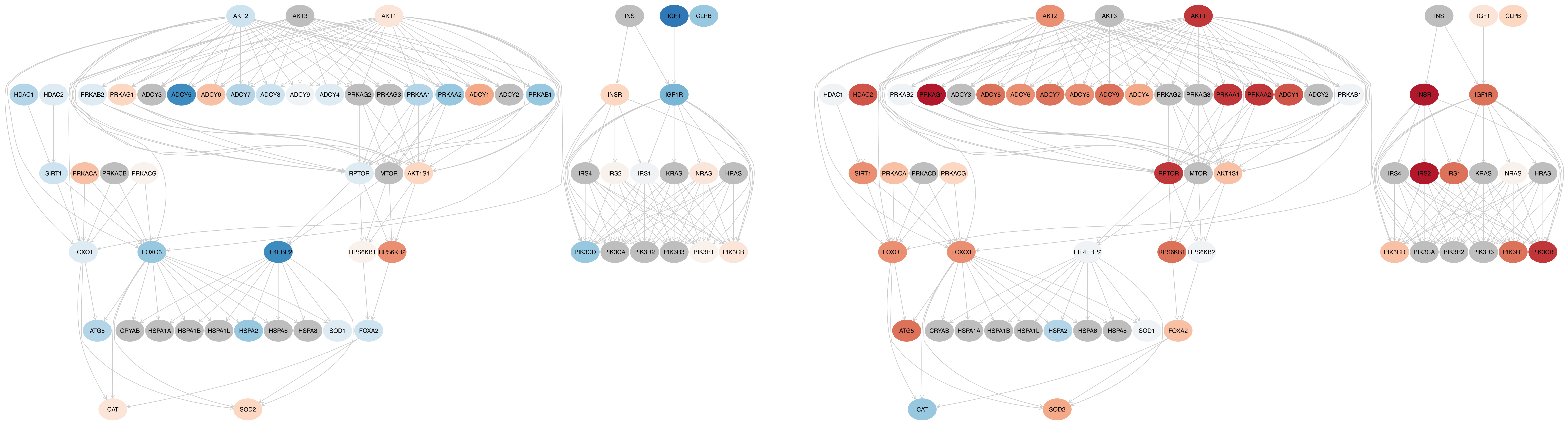

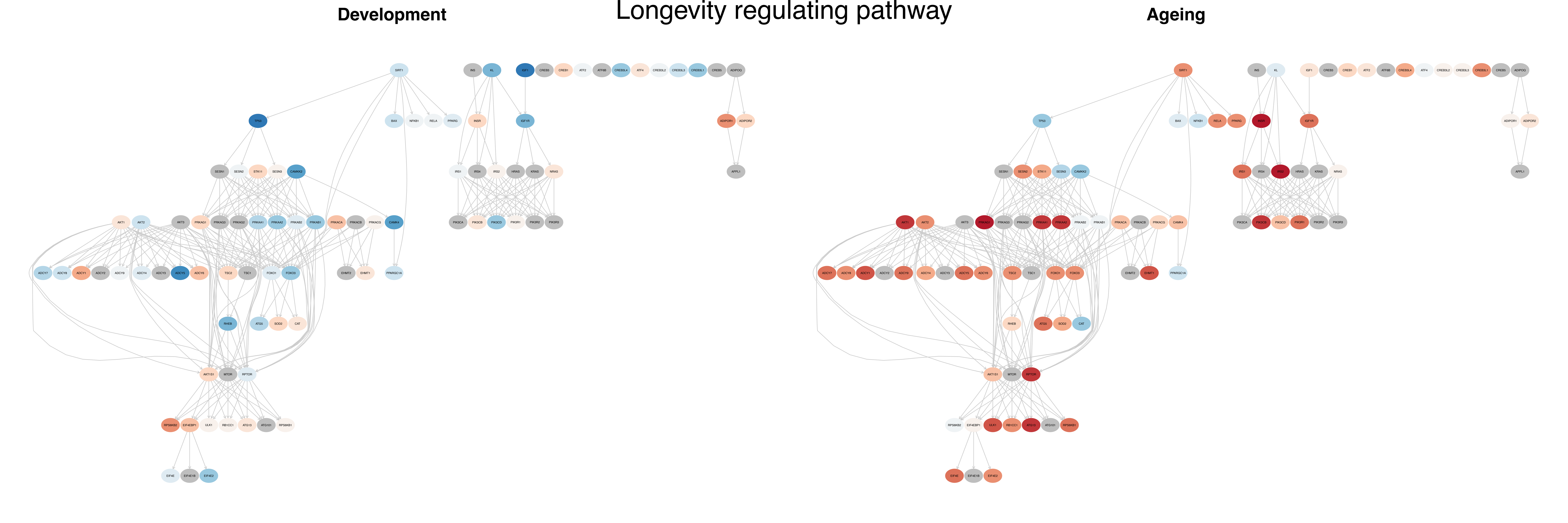
Longevity regulating pathway
Development
Ageing

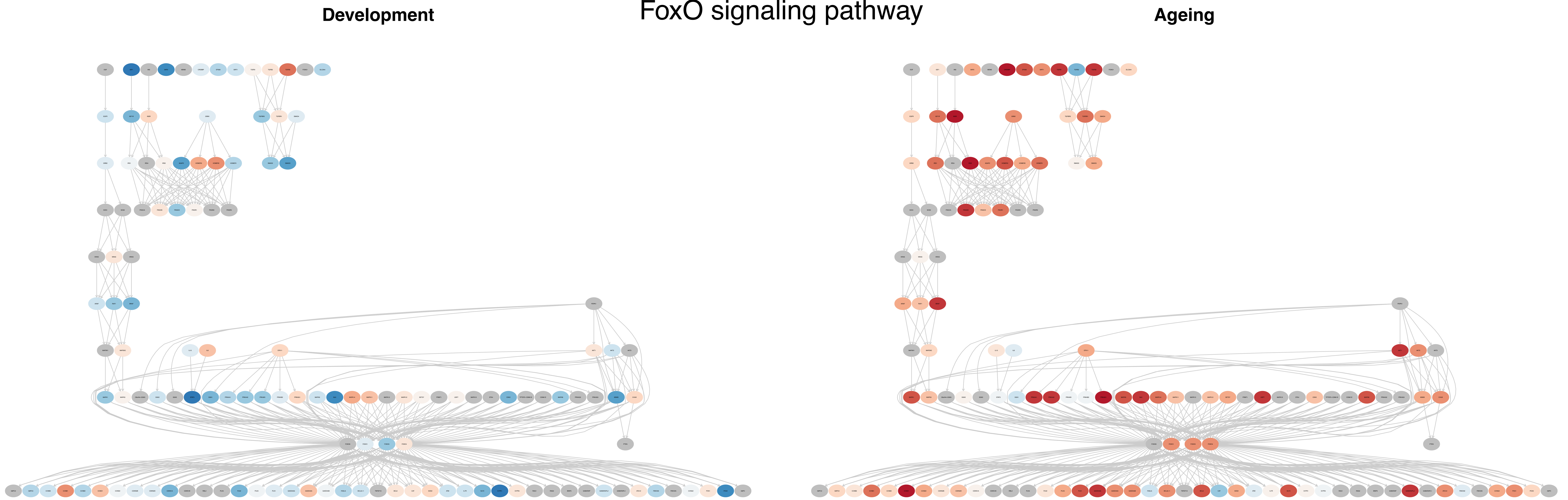

FoxO signaling pathway
Development
Ageing

Autophagy – animal

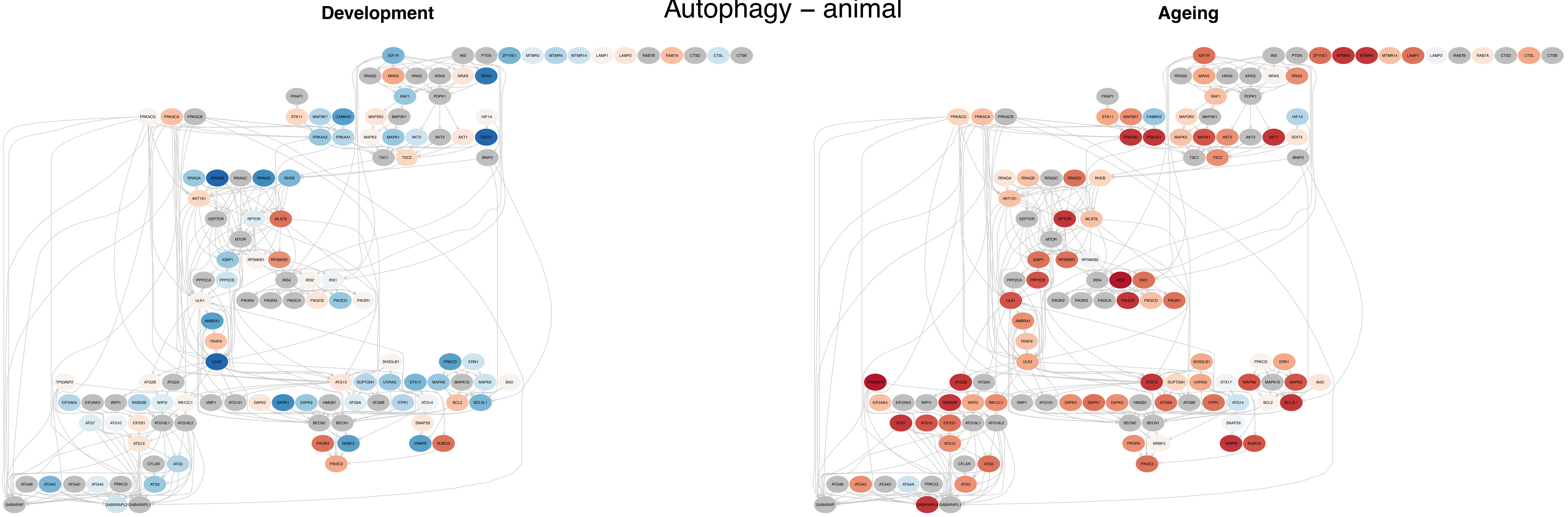

Adherens junction

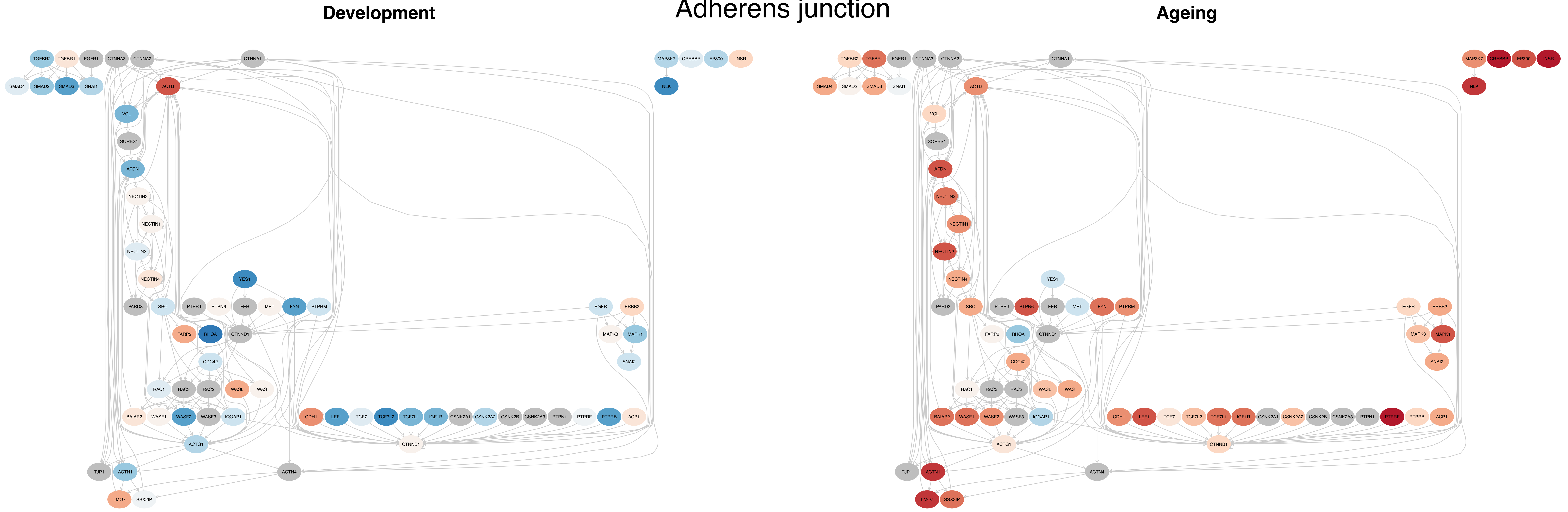

Apelin signaling pathway

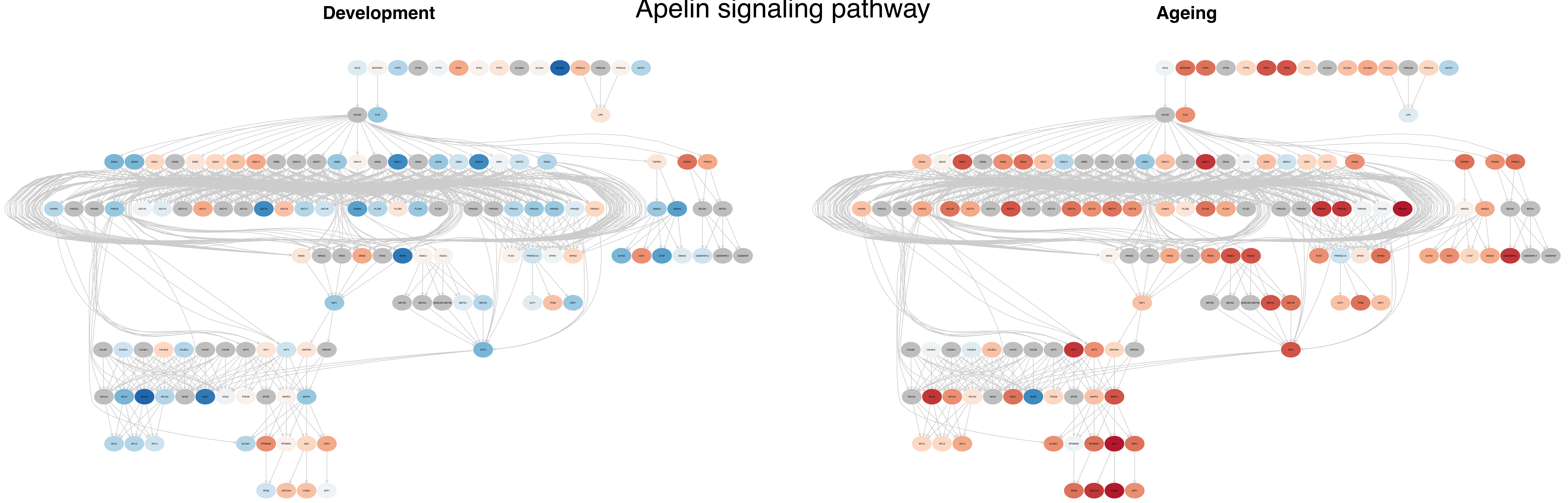

mTOR signaling pathway

Development **Ageing**

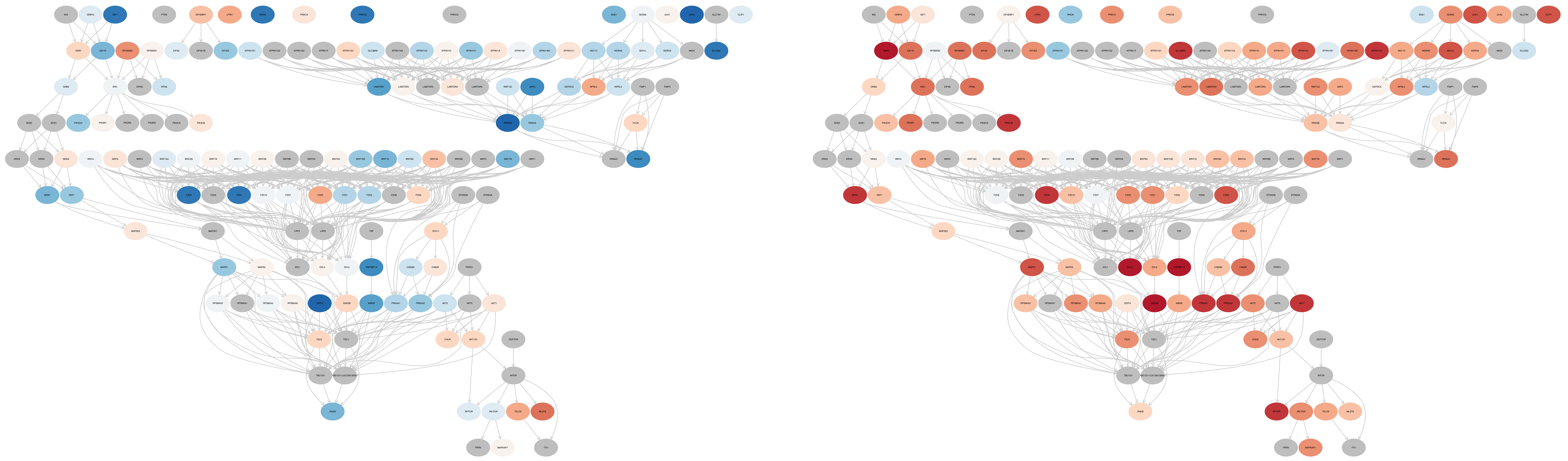

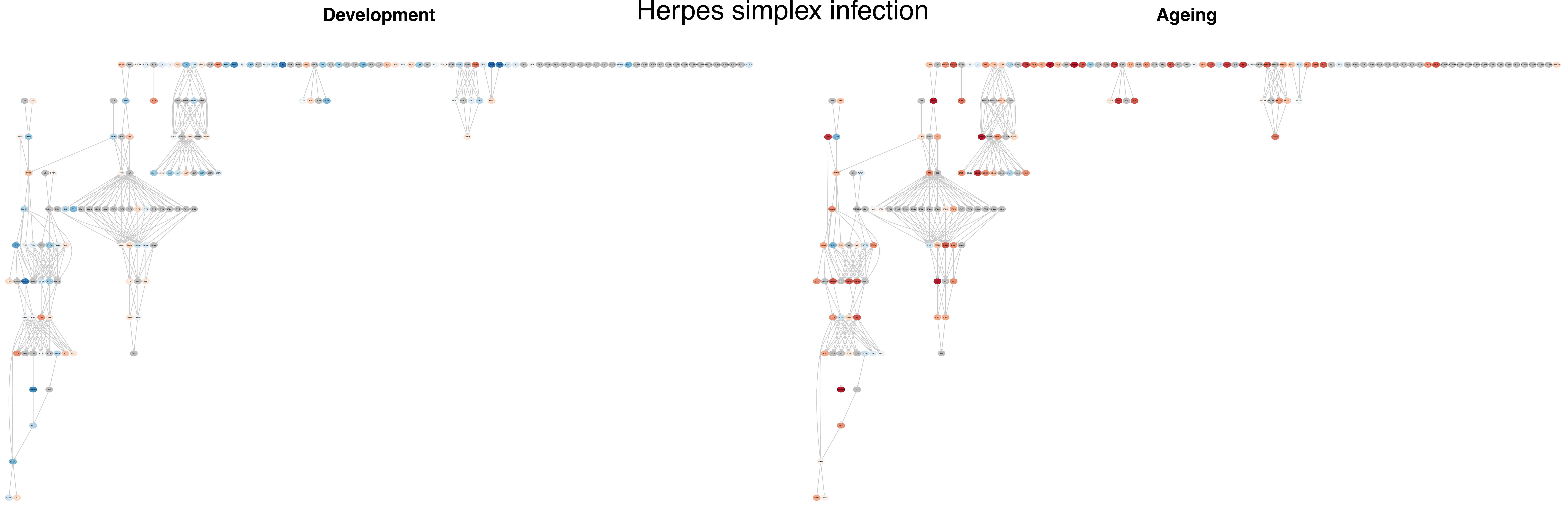
Herpes simplex infection
Development
Ageing

Axon guidance

Development **Ageing**

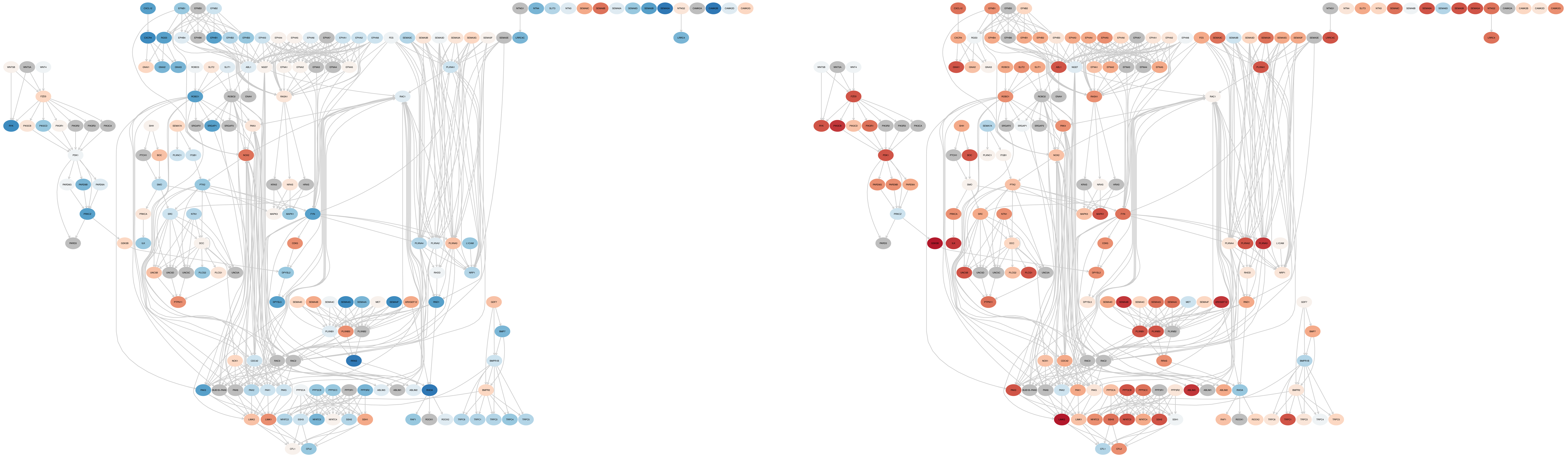

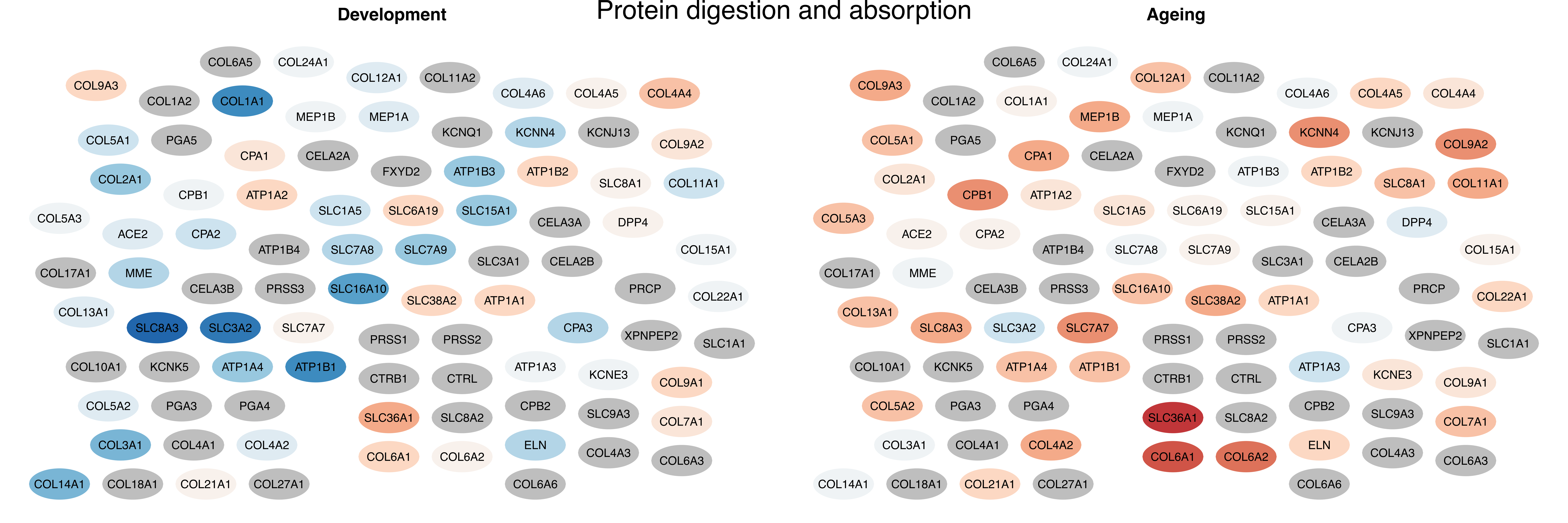
Protein digestion and absorption
Development
COL6A5
COL24A1
COL9A3
COL12A1
COL11A2
COL1A2
COL1A1
COL4A6
COL4A5
COL4A4
MEP1B
MEP1A
COL5A1
PGA5
KCNQ1
KCNN4
KCNJ13
CPA1
CELA2A
COL9A2
FXYD2
ATP1B3
ATP1B2
COL2A1
SLC8A1
COL11A1
CPB1
ATP1A2
SLC1A5
SLC6A19
SLC15A1
COL5A3
CELA3A
DPP4
ACE2
CPA2
ATP1B4
SLC7A8
SLC7A9
COL15A1
SLC3A1
CELA2B
COL17A1
MME
CELA3B
PRSS3
SLC16A10
PRCP
SLC38A2
ATP1A1
COL22A1
COL13A1
SLC8A3
SLC3A2
SLC7A7
CPA3
XPNPEP2
PRSS1
PRSS2
SLC1A1
COL10A1
KCNK5
ATP1A4
ATP1B1
ATP1A3
CTRB1
CTRL
KCNE3
COL9A1
COL5A2
PGA3
PGA4
CPB2
SLC36A1
SLC8A2
SLC9A3
COL7A1
COL3A1
COL4A1
COL4A2
ELN
COL6A1
COL6A2
COL4A3
COL6A3
COL14A1
COL18A1
COL21A1
COL27A1
COL6A6
Ageing
COL6A5
COL24A1
COL9A3
COL12A1
COL11A2
COL1A2
COL1A1
COL4A6
COL4A5
COL4A4
MEP1B
MEP1A
COL5A1
PGA5
KCNQ1
KCNN4
KCNJ13
CPA1
CELA2A
COL9A2
FXYD2
ATP1B3
ATP1B2
COL2A1
SLC8A1
COL11A1
CPB1
ATP1A2
SLC1A5
SLC6A19
SLC15A1
COL5A3
CELA3A
DPP4
ACE2
CPA2
ATP1B4
SLC7A8
SLC7A9
COL15A1
SLC3A1
CELA2B
COL17A1
MME
CELA3B
PRSS3
SLC16A10
PRCP
SLC38A2
ATP1A1
COL22A1
COL13A1
SLC8A3
SLC3A2
SLC7A7
CPA3
XPNPEP2
PRSS1
PRSS2
SLC1A1
COL10A1
KCNK5
ATP1A4
ATP1B1
ATP1A3
CTRB1
CTRL
KCNE3
COL9A1
COL5A2
PGA3
PGA4
CPB2
SLC36A1
SLC8A2
SLC9A3
COL7A1
COL3A1
COL4A1
COL4A2
ELN
COL6A1
COL6A2
COL4A3
COL6A3
COL14A1
COL18A1
COL21A1
COL27A1
COL6A6

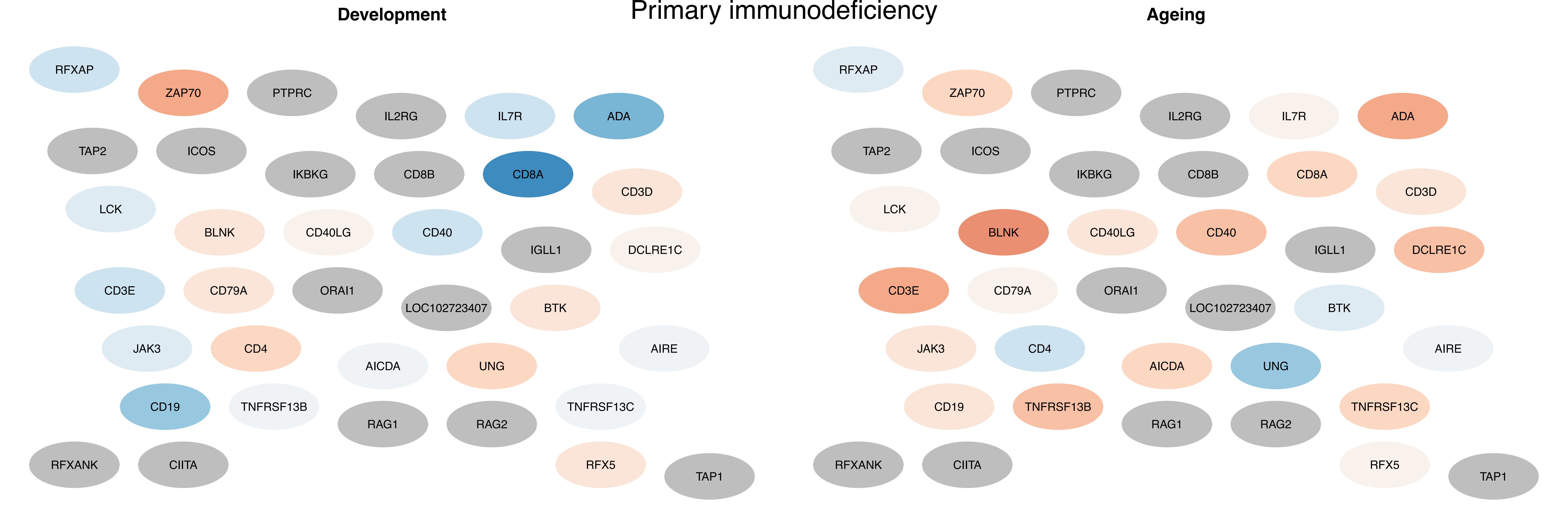
Primary immunodeficiency
Development
Ageing
RFXAP
ZAP70
PTPRC
IL2RG
IL7R
ADA
TAP2
ICOS
IKBKG
CD8B
CD8A
CD3D
LCK
BLNK
CD40LG
CD40
IGLL1
DCLRE1C
CD3E
CD79A
ORAI1
LOC102723407
BTK
JAK3
CD4
AICDA
UNG
AIRE
CD19
TNFRSF13B
RAG1
RAG2
TNFRSF13C
RFXANK
CIITA
RFX5
TAP1

Linoleic acid metabolism

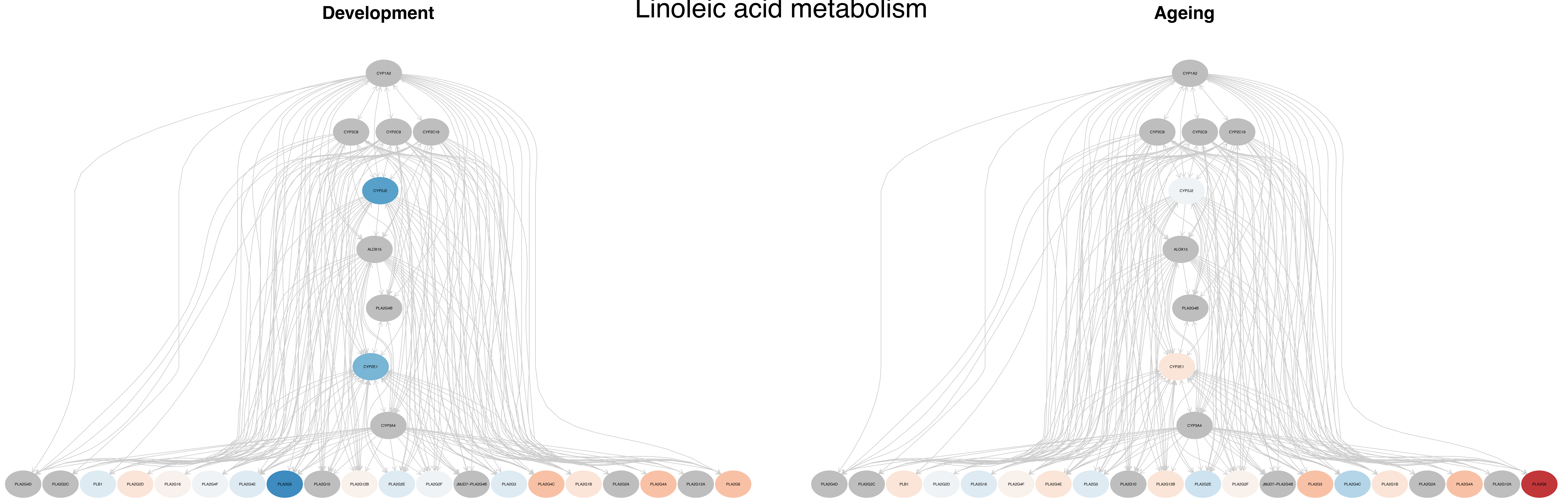

Fat digestion and absorption

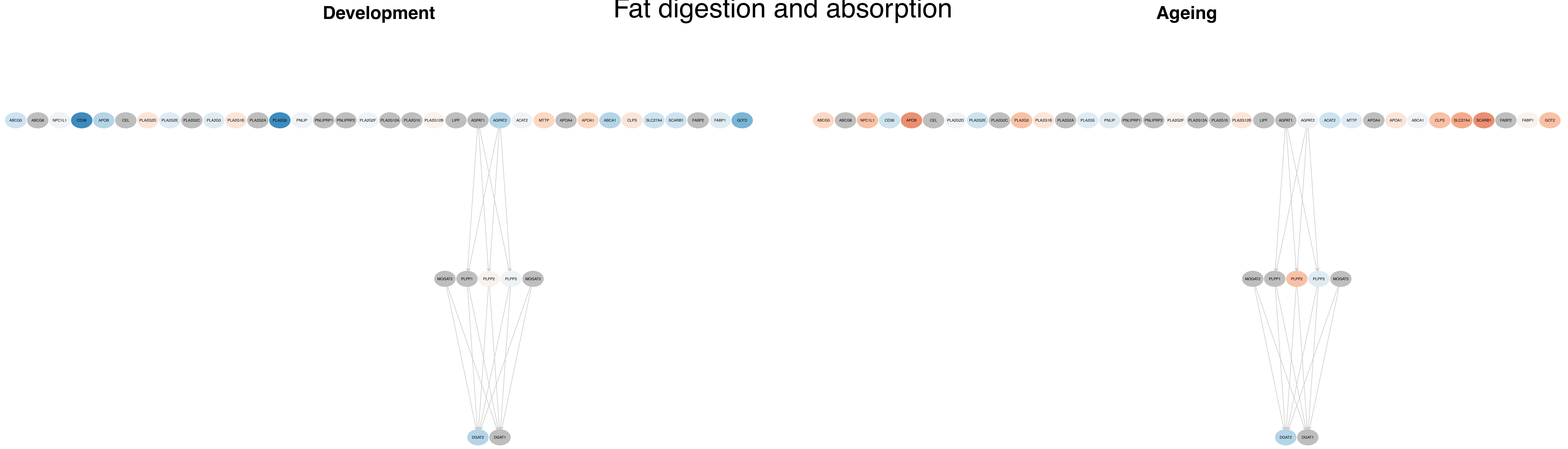

Supplement: Supplementary file 1 — Supplementary Information. [file 41598_2020_60998_MOESM1_ESM.pdf]
